# The opposite effects of stringent response on phage infection of *Pseudomonas putida*

**DOI:** 10.1093/femsml/uqaf048

**Published:** 2026-01-02

**Authors:** Alicja Cecylia Lewańczyk, Mariliis Hinnu, Elise Mägi, Roger Rikberg, Age Brauer, Hedvig Tamman

**Affiliations:** Department of Genetics, Institute of Molecular and Cell Biology, University of Tartu, Tartu 51010, Estonia; Department of Genetics, Institute of Molecular and Cell Biology, University of Tartu, Tartu 51010, Estonia; Department of Genetics, Institute of Molecular and Cell Biology, University of Tartu, Tartu 51010, Estonia; Department of Genetics, Institute of Molecular and Cell Biology, University of Tartu, Tartu 51010, Estonia; Department of Bioinformatics, Institute of Molecular and Cell Biology, University of Tartu, Tartu 51010, Estonia; Department of Genetics, Institute of Molecular and Cell Biology, University of Tartu, Tartu 51010, Estonia

**Keywords:** (p)ppGpp, stringent response, *Pseudomonas putida*, membrane defects, bacteriophages

## Abstract

Guanosine tetra- and pentaphosphate ((p)ppGpp) are one of the key players in the stress response of bacteria. Accumulation of these alarmones activates the stringent response, usually triggered by different nutritional stresses. For *Pseudomonas putida*, there is only limited data available on the importance of the stringent response in stress situations. Also, in recent years, different specific phage defence systems have received much attention, but little is known about the involvement of stringent response in phage infection. Here, we show that *P. putida* PaW85 (p)ppGpp^0^ is prototrophic and tolerates chemical stress well. However, in the stationary phase *P. putida* cells deprived of (p)ppGpp have impaired membrane integrity. In addition, we conducted a large-scale screening of stringent response effects on phage infections using the CEPEST phage collection. We tested 67 phages of 22 different species and revealed that the lack of (p)ppGpp has opposing effects on phage infection with nearly half of the tested phages showing higher infection efficiency on the (p)ppGpp^0^ cells, whereas the other half shows reduced infection. We show that the differences in phage infection efficiency for phages Aura and Amme-3 are not caused by adsorption rate differences, but alterations in downstream steps of the infection cycle—prolonged latent period in the absence of (p)ppGpp or unproductive infection in the presence of (p)ppGpp. Altogether, results indicate that the role of stringent response in phage infection is highly diverse, and over half of the times the presence of (p)ppGpp facilitates phage infections rather than protects the cells.

## Teaser

We demonstrate that some phages are inhibited by stringent response while others require it for efficient infections of *Pseudomonas putida*, a biotechnologically important model organism.

## Introduction

Bacteria encounter various stressful conditions during their life and need to respond efficiently in order to survive. The stringent response is a universal stress response activated by the accumulation of the alarmone molecules guanosine tetraphosphate (ppGpp) and guanosine pentaphosphate (pppGpp), collectively referred to as (p)ppGpp. The small nucleotide alarmones are produced by the RSH enzymes—RelA and SpoT homologs, named for two long (p)ppGpp synthesis/hydrolysis enzymes in *Escherichia coli* and other γ-proteobacteria (Atkinson et al. 2011). In γ- and α-proteobacteria, RelA is the main (p)ppGpp synthesis enzyme and has lost hydrolysis activity, whereas SpoT hydrolyses (p)ppGpp and has only a slight synthesis activity (Atkinson et al. 2011). In some bacteria, e.g.*Acinetobacter baumannii*, SpoT has completely lost its synthesis activity and functions solely as a hydrolase (Tamman et al. 2023). The deletion of the *spoT* gene in a *relA^+^* background is lethal to the bacteria as it results in an uncontrolled accumulation of (p)ppGpp (Xiao et al. 1991, Raskin et al. 2007). Besides long, multidomain RSH enzymes, some bacteria also code for single-domain proteins for (p)ppGpp synthesis and hydrolysis called small alarmone synthetases (SAS) or hydrolases (SAH), respectively (Atkinson et al. 2011). *Pseudomonas putida* PaW85 [isogenic to KT2440; Rosendahl et al. (2020)] belongs to the γ-proteobacteria group, and like *E. coli*, has the two long RSH enzymes, RelA (encoded by gene *PP_1656*) and SpoT (gene *PP_5302*) (Winsor et al. 2016). Additionally, *P. putida* KT2440 (PaW85) is predicted to encode one single-domain RSH protein, a SAH encoded by *PP_2450* (Atkinson et al. 2011), but it is annotated as a hypothetical protein of unknown function and localization in the Pseudomonas database (Winsor et al. 2016).

Upon stress, RSH enzymes activate to produce (p)ppGpp that reprograms bacterial physiology from growth to survival. The alarmone regulates various processes in *E. coli* cells, including replication, transcription, and translation [reviewed in Irving et al. (2021)]. The targets of (p)ppGpp and the mechanisms of the regulation of bacterial physiology have been a subject of research since its discovery in 1960s by Cashel and Gallant (1969). The stringent response is activated upon several stresses, such as amino acid (Haseltine and Block 1973), carbon (Flärdh et al. 1994), fatty acid (Seyfzadeh et al. 1993, Battesti and Bouveret 2006), iron (Vinella et al. 2005), or phosphate limitations (Spira et al. 1995), and also upon switching to the stationary phase (Lazzarini et al. 1971). Thus, activation of the stringent response helps cells survive many nutritional challenges. In addition, the levels of (p)ppGpp change upon temperature shifts (Mackow and Chang 1983, English et al. 2011). Upon (p)ppGpp accumulation cells show increased resistance and tolerance to different antimicrobials (Vinella et al. 1992, Hugonnet et al. 2016), and these decrease if the cells are incapable of producing (p)ppGpp (Gaca et al. 2013, Salzer and Wolz 2023). Thus, the stringent response helps bacteria overcome other external stresses besides nutritional limitations.

One challenge that bacteria commonly face is attack by bacteriophages (phages), viruses that infect bacteria. Phages thrive in all ecosystems where bacteria can be found (Chibani-Chennoufi et al. 2004), which makes these viruses a high threat for bacteria. Although quite a lot is known about the stringent response helping to overcome different stressful conditions [reviewed in Potrykus and Cashel (2008), Irving et al. (2021), Salzer and Wolz (2023)], not much is known about how the stringent response affects phage infections. For some phages, the lytic cycle is induced in (p)ppGpp-deficient conditions, and burst sizes and/or phage plaque size can be increased upon infection of cells that cannot produce (p)ppGpp (Nowicki et al. 2013, Patterson-West et al. 2018). Another report shows that the expression of (p)ppGpp synthesis genes *rel* and *relP* (encoding for a long RSH enzyme and SAS protein, respectively) is increased upon phage infection in *Staphylococcus aureus* (Fernández et al. 2017). Moreover, (p)ppGpp accumulation during infection of T7 coliphage has also been observed (Tabib-Salazar et al. 2018). Nevertheless, no large-scale analysis has been conducted, and moreover, the stringent response effect on phage infection in *Pseudomonads* has largely been overlooked.

Only limited data have been published about the stringent response of *P. putida*. In these bacteria, biofilm production and purine biosynthesis pathways have been shown to be affected by the stringent response (Sze et al. 2002, Díaz-Salazar et al. 2017, Vogeleer and Létisse 2022). Interestingly, while *E. coli* cells unable to produce (p)ppGpp are auxotrophic and require supplementation of several amino acids in order to survive (Xiao et al. 1991, Gentry and Cashel 1996), *P. putida* is prototrophic and able to grow on minimal media lacking additional amino acids (Sze et al. 2002). As *P. putida* and *E. coli* contrast in their ability to tolerate (p)ppGpp^0^ conditions (all (p)ppGpp synthesis enzymes removed from the genome), it is probable that the (p)ppGpp effects on the metabolic activity and cell physiology are different in *P. putida* compared to *E. coli*.

To better understand the effects of the stringent response to the physiology and stress tolerance of *P. putida* and understand the involvement of the stringent response in phage resistance, we created a (p)ppGpp^0^ strain of our laboratory strain PaW85 by deleting the chromosomal *relA* and *spoT* genes. We tested its viability, growth ability, and membrane integrity, and finally screened through the CEPEST collection of phages (Brauer et al. 2024) to determine the effects that the lack of stringent response may have on phage infection efficiency. We show that although the (p)ppGpp^0^  *P. putida* PaW85 cells are prototrophic and have only slightly decreased tolerance to different chemical stressors, the membrane damages caused by the lack of (p)ppGpp lead to lysis at late stationary phase growth. We also show that the absence of (p)ppGpp in the cells can have both facilitating or inhibiting effect on phage infections, but these changes are most likely not explained by the membrane defects of the (p)ppGpp^0^ cells, but rather changes in bacterial biosynthesis capabilities leading to alternative phage infection cycles.

## Methods

### Bacterial strains, media, and growth conditions

The bacterial strains, plasmids, and phages used in this work are listed in Table S1. The strains constructed during the study are all derivatives of *P. putida* PaW85 (Bayley et al. 1977), isogenic to KT2440 (Regenhardt et al. 2002). Bacteria were grown in lysogeny broth (LB, 1% tryptone, 0.5% yeast extract, 0.5% NaCl) or M9 minimal media (42 mM KH_2_PO_4_, 24 mM Na_2_HPO_4_, 19 mM NH_4_Cl, 9 mM NaCl) supplemented with microelement solution (with final concentration of 667 μM MgO, 50 μM CaCO_3_, 40 μM FeSO_4_, 12.5 μM ZnSO_4_, 12.5 μM MnSO_4_, 2.5 μM CuSO_4_, 2.5 μM, CoSO_4_, 1.9 μM H_3_BO_4_). The solid media contained 1.5% (w/v) and the soft agar 0.3% (w/v) of agar.

For selection, the growth media was supplemented with kanamycin (50 µg/mL) for *E. coli* and benzylpenicillin (Bp, 1500 µg/mL) and kanamycin (50 µg/mL) for *P. putida. Escherichia coli* was incubated at 37°C and *P. putida* at 30°C, except for phage experiments that were conducted at 20°C due to optimal infection conditions of the CEPEST phages (Brauer et al. 2024). For transformation of plasmids, the bacteria were electrotransformed according to the protocol of Sharma and Schimke (1996). Bacteria were stored in 30% glycerol stocks at −80°C. Agar plates were stored at 4°C up to 1 week. SM buffer (100 mM NaCl, 8 mM MgSO_4_, 50 mM Tris-HCl (pH 7.5), 0.01% gelatin) was used for storage and dilution of phages. Phages were stored at 4°C and in 30% glycerol stocks at −80°C.

### Construction of plasmids and strains

*Pseudomonas putida* ΔrelA, ΔrelAΔspoT, Δ4φΔrelA, and Δ4φΔrelAΔspoT strains were constructed by sequential deletion of *relA* and *spoT* genes from *P. putida* wild-type PaW85 and its Δ4φ derivative (Brauer et al. 2024). The *relA* gene was always deleted first and *spoT* gene from the derived ΔrelA strain to prevent accumulation of (p)ppGpp in the ΔspoT strain. The pEMG-based plasmids were used for strain construction according to a well-described protocol (Martínez-García and de Lorenzo 2011). Oligonucleotides used in PCR amplifications are listed in Table S2. For the deletion of *relA* and *spoT* genes, the upstream and downstream regions of corresponding genes were amplified separately with primer pairs XbaRelA-For/RelAdel-Rev and RelAdelLONG-For/RelAEcoRI-Rev for deletion of *relA* and XbaSpoT-For/SpoTdel-Rev and SpoTdelLONG-For/EcoRI SpoT-Rev for deletion of *spoT*, respectively. Two PCR products were joined into one, ~1-kbp fragment by overlap extension PCR using the pairs of primers XbaRelA-For/RelAEcoRI-Rev and XbaSpoT-For/EcoRI SpoT-Rev, respectively, for *relA* and *spoT* and inserted into a pEMG plasmid (Martínez-García and de Lorenzo 2011) opened with XbaI and EcoRI. The genomes of all final strains were subsequently sequenced.

### Whole genome sequencing and analysis

The genomes of each deletion strain were extracted from cells grown 16 h at 30°C using GeneJET genomic DNA purification kit (Thermo Fisher Scientific) according to the manufacturer’s protocol. Deletion strains were sequenced using the Illumina NextSeq2000 platform with paired-end 2 × 151 bp reads. Reads were cleaned and filtered with fastp (Chen et al. 2018). Presence of deletions and lack of other potentially relevant mutations were confirmed with breseq v.0.35.0 (Deatherage and Barrick 2014) using *P. putida* KT2440 (NC_002 947) as a reference.

### Growth assays

For preculture, bacteria were diluted 50-fold from overnight cultures (ONCs) into 5 mL of fresh LB medium and grown with shaking. For carbon source growth assays, M9 minimal medium (Sambrook and Russell 2001) was used with a 0.2% final concentration of carbon source (glucose, gluconate, glycerol, succinate, or fructose) ±0.2% cas-amino acids with an additional 10 µg/mL tryptone (CAA). For all M9 growth assays, cells were first pelleted (1 min, 12 100 g), the LB medium was removed, and bacteria were resuspended in M9 solution without a carbon source. For liquid growth assays, exponential cells were diluted to an OD_580_ 0.1 in LB or M9 solution without a carbon source. 50 µL of OD_580_ 0.1 culture was added to microtiter plate containing 50 µL of medium. Growth was recorded in a plate reader (BioTek Synergy H1) with continuous shaking. Doubling times were calculated based on OD_580_ curves from exponential growth phase for each media according to the following formulas: (i) growth rate =$\frac{{\ln ( {OD1} ) - \ln ( {OD2} )}}{{( {t2 - t1} )}}$ and (ii) doubling time = $\frac{{\ln ( 2 )}}{{\textit{growth}\ \textit{rate}}}$. For colony forming unit (CFU) counts, precultures were grown for indicated time, and final CFUs were determined from 5 µL spots of 10-fold dilutions in 0.9% saline or M9 without carbon source after overnight (ON) incubation on agar medium. For M9 plate assays, only stationary phase cultures were studied. All media were prewarmed, and incubations were carried out at 30°C.

### Chemical growth inhibition assays

Minimum inhibitory concentrations (MICs) to various chemicals were determined in LB medium following general recommendations for microdilution susceptibility testing assay (European Committee for Antimicrobial Susceptibility Testing (EUCAST) 2003). For preculture, bacteria were diluted 50-fold from ONC into 5 mL fresh LB medium. Cells were grown to exponential phase with shaking and diluted to ∼10^5^ CFU/mL starting concentration on a microtiter plate with serially diluted chemicals in 100 µL final volumes. MICs were recorded after static incubation in a humid environment for 18–20 h. For liquid growth curve assays, precultures were prepared in the same way, however, the plates were incubated in a plate reader (BioTek Synergy H1) with shaking. All media were prewarmed, and incubations were carried out at 30°C.

For LB-agar plate assays, chemicals were added to liquid LB (<60°C) containing 1.5% agar. 20 mL of medium was poured into each Petri dish. Precultures were grown for 24 h in LB and serially diluted in 0.9% saline. 5 µL of each dilution was spotted on plates, dried, and incubated at 30°C. Plates were scanned after one and two ON incubations at 30°C. Experiments were done in at least three biological replicates.

### Microscopy for cell size assessment

For preculture, bacteria were diluted 50-fold from ONCs into 5 mL of fresh LB medium and grown with shaking. Bacterial cultures at different growth states were washed once with sterile-filtered 0.9% saline and imaged with wide-field microscopy (Zeiss Axio Observer Z1) on 1% agarose pads (1:1 mix of low-melt:high-melt agarose) in phosphate-buffered saline. Cell sizes were determined from microscopy images with ImageJ software tool MicrobeJ (Ducret et al. 2016). Statistical analysis was carried out with Prism Software (GraphPad).

### Flow cytometry

Syto 9 and propidium iodide (PI) (Thermo Fisher Scientific) staining of bacterial cells was used to assess cell membrane permeability. Bacterial cultures at different growth states were washed once with filter-sterilized 0.9% saline and stained with a final concentration of 7.5 µM Syto 9 and 30 µM PI for at least 15 min in the dark at room temperature. Fluorescence intensities were recorded with flow cytometry (BD FACS Aria) (Syto9 λ_ex_ 488 nm/λ_em_ 530/30 nm; PI λ_ex_ 488 nm, λ_em_ 616/23). 6 µm diameter beads (Thermo Fisher Scientific) were added to flow cytometry samples to estimate the sample volume. Fluorescent dyes DiOC_2_(3) (3,3'-Diethyloxacarbocyanine Iodide; final concentration 30 µM) and DiSC_3_(5) (3,3'-Dipropylthiadicarbocyanine Iodide; final concentration 5 µM) (Thermo Fisher Scientific) were used to estimate membrane potential. Carbonyl cyanide m-chlorophenyl hydrazone (CCCP) (Thermo Fisher Scientific) was used as a membrane depolarizing control. Bacterial cultures at different growth states in LB medium were diluted into either of the dye solutions, using 1X M9 salt solution as diluent, for at least 15 min at room temperature. Fluorescence intensities were recorded with flow cytometry (BD FACS Aria) (green fluorescence λ_ex_ 488 nm/λ_em_ 530/30 nm; red fluorescence λ_ex_ 633 nm, λ_em_ 660/20).

Flow cytometry samples were analysed with FlowJo™ Software version 10.7 (BD Life Sciences). Gating strategy is shown in Fig. S1. Statistical analysis was carried out with Prism Software (GraphPad).

### Full proteome sample preparation and analysis

For preculture, bacteria (*P. putida* PaW85 wild-type and ΔrelAΔspoT) were diluted 50-fold from ONC into 5 mL fresh LB medium. Cells were grown to exponential phase (OD_580_ ∼1) with shaking. Cells were washed once with 1X M9 salt solution and diluted to OD_580_ 0.05 in either LB or M9 minimal medium with 0.2% glucose or succinate in 5 mL volumes. Cells were cultured with shaking for 24 h. All media were prewarmed, and incubations were carried out at 30°C. Three biological replicates were analyzed in each condition. For label-free proteomics, 1 mL of culture was washed once with prewarmed 1X M9 salt solution, supernatant was removed, and the dry pellet was flash-frozen in liquid nitrogen. Samples were stored at –80°C until analysis at The Proteomics Core Facility of University of Tartu.

### Liquid chromatography—tandem mass-spectrometry analysis (LC-MS/MS)

Cell pellets were resuspended in lysis buffer (6 M Gu-HCl, 100 mM Tris-HCl pH 8.5, 50 mM DTT) and heated at 95°C for 5 min. After cooling, the lysates were homogenized by sonication in a Bioruptor (Diagenode) sonicator 10X of a cycle: 30 s ON, 60 s OFF, High setting at 4°C. Further homogenization was carried out with Silibeads TypZY-S 0.4–0.6 mm beads in a FastPrep24 bead beater device (MP Biomedicals), using 2 × 40 s 6 m/s pulses (cooling for 5 min between pulses). Resulting lysate was cleared by centrifugation at 17 000 × *g* for 10 min at 4°C. 10 µg of resulting proteins was precipitated with 20% TCA and 0.8 mg/mL sodium deoxycholate and washed with acetone and methanol. Dried protein pellets were resuspended in 7 M urea, 2 M thiourea, 100 mM ammonium bicarbonate, and 20 mM methylamine buffer, reduced (10 mM DTT for 30 min) and alkylated in dark (30 mM chloroacetamide for 30 min) before adding Lys-C protease (1:100 ratio over substrate; Fujifilm Wako Pure Chemical, Osaka, Japan) for predigestion (25°C for 2 h). Samples were diluted 1:5 with 100 mM ammonium bicarbonate before adding dimethylated proteomics grade porcine trypsin (Sigma Aldrich) at 1:50 ratio over substrate and incubating for 16–18 h at 25°C. Digested samples were acidified with 1% trifluoroacetic acid and desalted using in-house prepared C18 (3 M) solid phase extraction tips.

LC-MS/MS analysis setup involved Dionex Ultimate 3000 RSLCnano reverse chromatography system connected to Q Exactive HF (Thermo Fisher Scientific) mass spectrometer. Tryptic peptides were loaded onto a C18 trap-column (0.3 × 5 mm; 5 µm particles, Dionex) and injected into an in-house packed (3 µm C18; Dr Maisch, Ammerbuch, Germany) analytical emitter-column (50 cm × 75 µm; MS Wil, CoAnn Technologies, the Netherlands.) 90 min 5%–35% buffer B gradient was used to separate the peptides (buffer A: 0.1% formic acid; buffer B: 80% acetonitrile and 0.1% formic acid). Eluted peptides were delivered into a mass spectrometer via a nano-electrospray source, using positive mode and spray voltage of 2.5 kV. Each 350–1400 m/z full MS scan was followed by higher-energy collisional dissociation fragmentation (normalized collision energy set at 26) and MS/MS analysis of the 12 most intense ions. The settings used for MS and MS/MS analysis, respectively, were: resolution *R* = 60 000 and 30 000, the ion number target values 3 000 000 and 100 000, and the maximum injection times 50 and 41 ms. Ion charge for analysis was set *z* = +2 to +5, MS/MS isolation window to 1.6 m/z, and dynamic exclusion to 40 s.

The raw MS files were processed with the MaxQuant software package (version 2.2.0.0) (Cox and Mann 2008), using default search parameters. Methionine oxidation and protein N-terminal acetylation were set as variable modifications, and cysteine carbamidomethylation as a fixed modification. *Pseudomonas putida* reference proteome (downloaded in October 2024 from Uniprot archive) and a common contaminating proteins databases (Frankenfield et al. 2022) were used in the search.

Protein groups were analysed with Perseus software (version 2.1.5.0) (Tyanova et al. 2016). For statistics, student two-sample T-test was used with Benjamini-Hochberg multiple comparison and a false discovery rate of 0.05. Full list of differentially expressed genes in any of the tested conditions grouped by function can be found in Supplementary proteomics data file.

The mass spectrometry proteomics data have been deposited to the ProteomeXchange Consortium via the PRIDE (Perez-Riverol et al. 2025) partner repository with the dataset identifier PXD070597 and 10.6019/PXD070597.

### Phage infection

Bacteria were grown in LB medium overnight at 20°C. The ONCs were diluted 15-fold into fresh LB medium (5 mL) and grown at 20°C until OD_580_∼1 to obtain precultures.

For plate assays, bacteria were grown for 48 h, and 50 μL of the culture was mixed with 5 mL of melted LB soft agar medium (42°C) containing 10 mM CaCl_2_, and overlaid on LB agar plates to create a bacterial lawn. Phage stocks were then serially diluted in SM buffer, and 1.5 μL of each dilution was spotted on the bacterial lawn. Phage plaques were counted after incubating the plates overnight at 20°C. Experiments were done in three biological replicates.

For plaque size measurements, 200 μL of the bacterial preculture (OD_580_∼1) was mixed with phage stock, in adjusted concentration to obtain about 100–200 phage plaques per plate, 5 mL of LB soft agar medium (42°C) containing 10 mM CaCl_2_ and overlaid on LB agar plates to create a bacterial lawn. Plate scans were taken after incubating the plates overnight at 20°C. The diameter of phage plaques was measured with ImageJ software. Calculations were performed based on 100 phage plaque size measurements per sample.

For liquid assays, precultures (OD_580_∼1) were diluted to an OD_580_ 0.1 in fresh LB supplemented with 10 mM CaCl_2_, and phages were added to each culture at different MOI (multiplicity of infection, number of phages per one bacterium) values with the final volume of the mix of 100 μL. Growth following phage infection was measured by optical density (OD) measurements at 580 nm in 96-well plates at 10-min intervals (using the Magellan v7.1 software) at 20°C on a plate reader (Tecan Infinite M200 Pro). Liquid assay experiments were repeated three times with each containing at least six technical replicates. All phage infection data were analysed with Prism software (GraphPad).

### Phage adsorption

For adsorption measurements, bacterial preculture (OD_580_∼1) was supplemented with 10 mM CaCl_2_, then 250 μL of the preculture was mixed with 650 μL of LB supplemented with 10 mM CaCl_2_, diluting the bacteria to around 3 × 10^7^ CFU/mL, and 100 μL of phage was added at a MOI (multiplicity of infection) of 0.1. Samples of 100 μL were taken every 5 min (up to 20), added to 10 μL of chloroform, and kept on ice to inhibit further adsorption for a maximum of 1 min (until taking the last sample of each time-point). Then, samples were immediately centrifuged (12 100 × *g*, 45 s), the supernatant was serially diluted in SM buffer, and 1.5 μL of each dilution was spotted on the bacterial lawn of a sensitive strain. Phage plaques were counted after incubating the plates overnight at 20°C. Experiments were repeated in at least three biological replicates. Data were analysed using Prism software (GraphPad).

### One-step growth curves

The latent period and burst size of Amme-3 and Aura phages were determined by creating the one-step growth curves. 10 mM CaCl_2_ was added to the media when starting the preculture. When OD_580_ reached ∼1, 1250 μL of the preculture was mixed with 650 μL of LB supplemented with 10 mM CaCl_2_, and 100 μL of phage dilution was added to the final MOI of 0.1. After allowing phages to adsorb for 20 min, the mixtures were centrifuged (MiniSpin, rotor F-45–12-11, 12 100 × *g*, 2 min) and the supernatant was replaced with fresh LB to remove unbound phages. The cells with bound phages were then diluted 10 times in LB with 10 mM CaCl_2_ to minimize secondary adsorptions. The amount of phages in the mix was determined by removing 100 μL of sample at certain time points (every 15 min up to 120 min for Amme-3; and every 30 min up to 330 min for Aura). The samples were serially diluted in SM buffer and 1.5 μL of each dilution was spotted on the bacterial lawn of a sensitive strain. Phage plaques were counted after incubation at 20°C overnight.

### Phage labelling and microscopy

Amme-3 phage particles were labeled with Syto 13 Green Fluorescent Nucleic Acid Stain (Thermo Fisher Scientific) according to protocol (Low et al. 2020). Shortly, a purified phage stock (titer > 10^10^ PFU/mL) was mixed with Syto 13 (final concentration 0.25 mM) for 15 min in the dark at room temperature. A 10-fold volume of SM buffer was added to the dyed phage and allowed to equilibrate for an additional 5 min. Free dye was removed by filtering slowly through a polyethersulfone (PES) membrane filter with a pore size of 0.45 µm, and the labeled phage was used immediately for infection. Bacteria were grown in LB medium (with 10 mM CaCl_2_) to the exponential phase (OD_580_ ∼1) at 20°C with shaking, concentrated 4X, mixed in equal volume with the labeled phage. To visualize the initial stages of infection (attachment of phages and injection of labeled phage DNA), 1 μL of the sample was put on a glass immediately after mixing bacteria with phages, and screened with a wide-field microscope (Olympus BX61 Fluorescence Microscope, 100× magnification) as a time course imaging in intervals up to 30 min. U-MNIBA3 filter (λ_ex_ 482.5/25 nm, λ_em_ 530/40 nm) was used for imaging Syto 13-tagged bacteriophages. To study the further stages of infection, a mix of bacteria and phages was left for adsorption for 30 min, the samples were centrifuged (MiniSpin, rotor F-45–12-11, 12, 100 × *g*, 1 min), the supernatant was replaced with half a volume of fresh LB (with 10 mM CaCl_2_) to remove unbound phages and concentrate the sample for imaging. Bacteria with labeled phages were imaged using wide-field microscopy on 1% agarose pads (1:1 mix of low-melt: high-melt agarose) in 1X M9 and 0.01X LB medium in 30-min intervals up to 240 min of infection, with new microscopy slides prepared for each timepoint. Microscopy images were analysed with Imaris software and ImageJ software with MicrobeJ tool.

## Results and discussion

### *Pseudomonas putida* PaW85 (p)ppGpp^0^ strain is prototrophic

Stringent response regulates a great number of different processes in bacterial cells. To see how the lack of stringent response influences the growth and physiology of *Pseudomonas putida* PaW85, we constructed two strains by genomic deletions. The first strain lacks the *relA* gene encoding the main (p)ppGpp synthesis enzyme RelA (ΔrelA) and the second strain lacks both *relA* and *spoT* genes (ΔrelAΔspoT) and is thus unable to produce the alarmone molecule (p)ppGpp ((p)ppGpp^0^). Then we set out to compare the growth of these strains to the wild-type bacteria.

**Figure 1.**
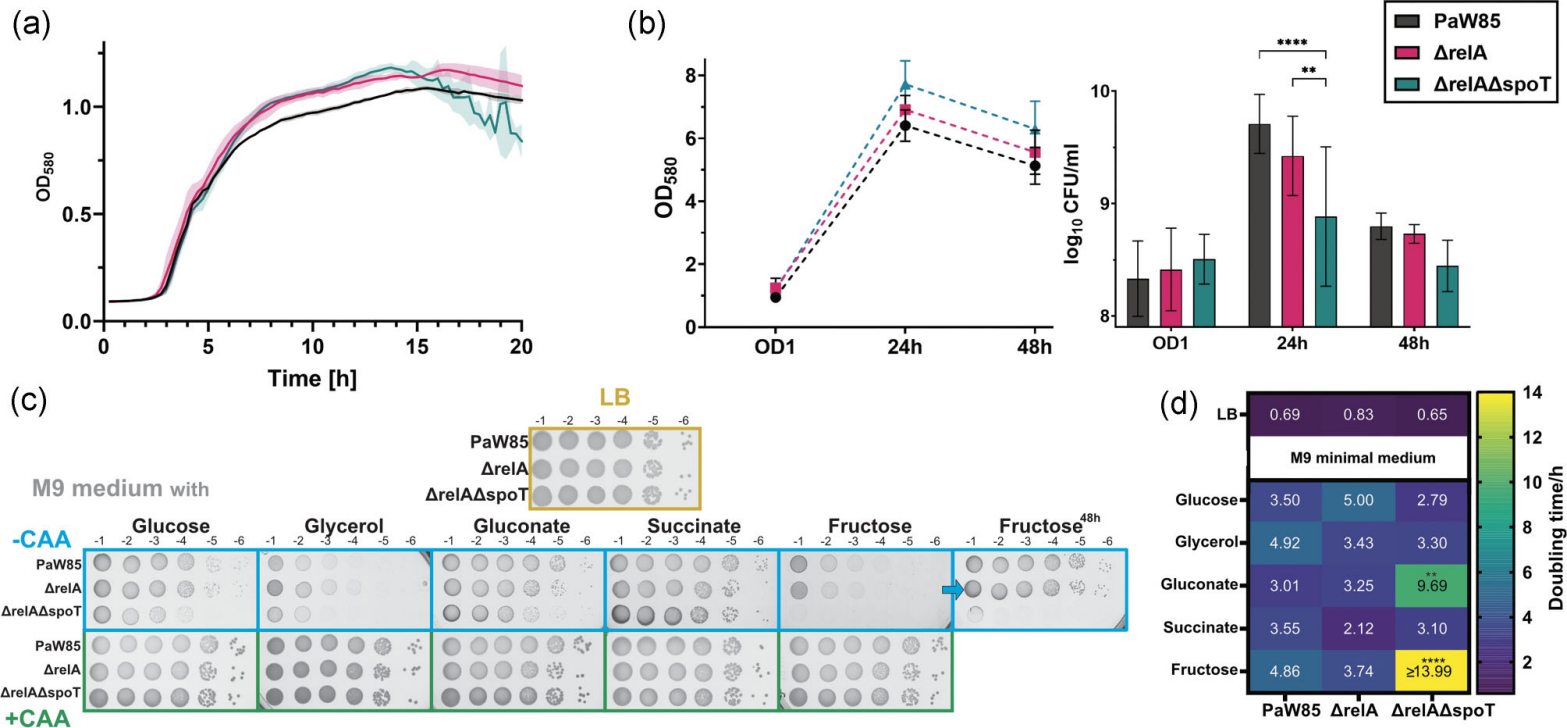
The growth of *P. putida* PaW85 wild-type, its main (p)ppGpp synthesis enzyme RelA deletion (ΔrelA) and (p)ppGpp^0^ (ΔrelAΔspoT) derivatives. Colors indicated on the legend. (a) Growth curves in LB medium measured with a microtiter plate reader. (b) The OD (580 nm) of bacterial culture and CFU measured from the same 5 mL cultures after growth to OD_580_ 1 (∼2.5 h), 24 h, and 48 h. (c) The growth of stationary cells on solid minimal M9 media (-CAA) with 0.2% carbon source or the same with added 0.2% casamino acids (+CAA). Plates were incubated at 30°C for 24 h before recording the result. (d) Mean doubling times (*N* = 3) in LB and M9 minimal media (-CAA) based on microtiter plate optical densities. Means ± SD (*N* ≥ 3) shown for (a) and (b). Statistical test with ordinary two-way ANOVA with Tukey’s multiple comparisons test with single pooled variance. **P-value < .005; ****P-value < .0001

In rich media (lysogenic broth, LB), both deletion strains (ΔrelA and ΔrelAΔspoT) grow well, and cultures reach densities comparable to the wild-type or even slightly higher (Fig. 1a, b). Nevertheless, in 24 h cultures, the number of CFUs are significantly decreased for the (p)ppGpp^0^ strain compared to wild-type (Fig. 1b), and the same tendency can also be observed for late stationary phase cultures grown for 48 h (Fig. 1b). We hypothesize that the discrepancy between OD and CFUs might be due to an increased number of dead cells in bulk culture and/or aggregation in the ΔrelAΔspoT strain.

On minimal media, the growth of the ΔrelA strain is similar to wild-type *P. putida* PaW85, suggesting that SpoT can compensate for the synthesis of (p)ppGpp in the absence of RelA. The (p)ppGpp^0^ strain ΔrelAΔspoT experiences decreased colony size when grown for 24 h at 30°C, except when provided succinate as the carbon source (Fig. 1c). Yet, on most of the tested carbon sources (all except fructose), the growth of (p)ppGpp^0^ strain after 2 days of incubation at 30°C looks similar to the other strains (wild-type and ΔrelA, Fig. S2A), suggesting a reduction in growth rate and not viability on solid minimal medium. It confirms that the (p)ppGpp^0^  *P. putida* PaW85 is prototrophic and does not require the addition of any amino acids for growth. The only carbon source severely inhibiting the growth of the (p)ppGpp^0^ strain is fructose without the addition of casamino acids (CAA).

We obtained similar results comparing the growth of the strains in liquid minimal media and observed that the growth curves between the wild-type and (p)ppGpp^0^ strains differ mainly by the maximum density reached (glucose, gluconate, succinate, Fig. S2B), but in all the tested media except fructose, the cells were able to grow even without the addition of CAAs (Fig. S2B). Doubling times were significantly longer with gluconate and fructose as a sole carbon source, whereas in glucose, glycerol and succinate, the (p)ppGpp^0^ strain had a shorter doubling time than the wild-type (Fig. 1d). In all media tested, the addition of CAA abolished any significant differences with rich LB (doubling times between 0.68 and 1.04 h across all media and strains) (Fig. S3).

These data show that (p)ppGpp^0^ PaW85 is prototrophic with slightly reduced growth without additional amino acids on most carbon sources tested. When CAA are added to the media, the growth of (p)ppGpp^0^ is similar to the wild-type bacteria both on the plates and in liquid media (Figs. 1c and S2) and even on fructose-containing medium.

The ability of *P. putida* KT2440 (p)ppGpp^0^ strain to grow in minimal media has been recorded previously (Sze et al. 2002). Nevertheless, we observe very different effects on growth depending on the carbon source provided. The growth of (p)ppGpp^0^ strain differs least of wild-type on succinate-containing media (Fig. 1c). Succinate enters the carbon metabolism pathway of *P. putida* the latest, being included directly into the tricarboxylic acid (TCA) cycle (Molina et al. 2019, Kanehisa et al. 2025). No growth reduction observed on succinate-containing media (Fig. 1c) suggests that the TCA cycle functions in a similar manner both with and without cellular (p)ppGpp. The other carbon metabolism pathways are probably affected by the lack of (p)ppGpp. Indeed, the upper parts of the Embden–Meyerhof–Parnas, Entner–Doudoroff pathway, and pentose phosphate pathways are (p)ppGpp dependent (Vogeleer and Létisse 2022) as the metabolites of these pathways are deregulated upon stringent response induction by serine hydroxamate in a (p)ppGpp dependent manner (Vogeleer and Létisse 2022). This suggests that although (p)ppGpp^0^  *P. putida* PaW85 is able to grow in minimal media, the alarmone molecule still plays an important role in regulating different carbon metabolism pathways of *P. putida*.

**Figure 2.**
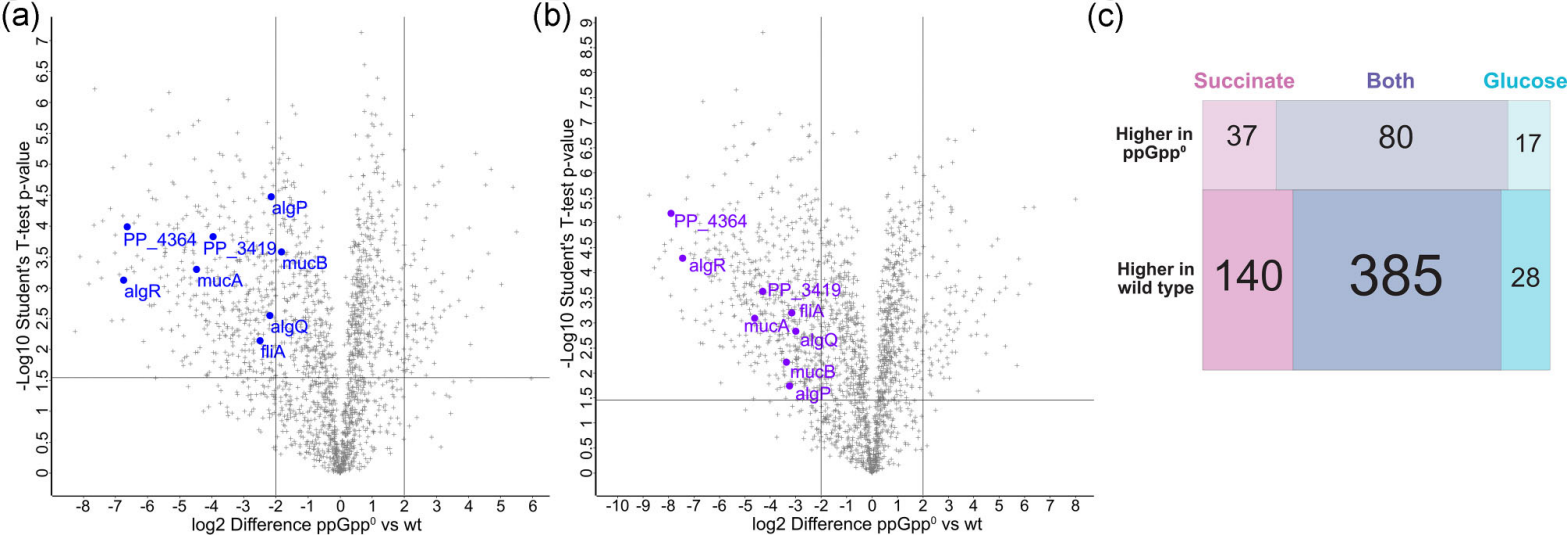
Full cell proteome analysis scatter plots of *P. putida* PaW85 (p)ppGpp^0^ derivative compared to the wild-type grown in minimal media with either glucose (a) or succinate (b) as the carbon source. Horizontal lines represent statistically significant values (student’s T-test with Benjamini-Hochberg multiple comparison with false discovery rate 0.05), vertical lines show four times under- or overexpression in (p)ppGpp^0^ strain compared to wild-type. The anti-σ^70^ factors AlgQR, MucAB, PP_3419 and PP_4364 and transcriptional regulator AlgP) are brought out in with labels (a, glucose and b, succinate). (c) Overall comparison of differentially expressed genes of (p)ppGpp^0^ strain and wild-type *P. putida* PaW85 grown in either succinate or glucose containing media.

To elucidate this effect, we decided to take a closer look into the gene expression patterns of *P. putida* PaW85 and its (p)ppGpp^0^ derivative grown in M9 media with either glucose or succinate as the carbon source. We conducted a whole cell proteome analysis to compare the gene expression of the two strains. As expected, the differences in the proteins produced by the two strains, are excessive (Fig. 2, Supplementary proteomics data). We could not, however see a clear difference in the expression of carbon metabolism pathways upon growth on neither glucose nor succinate (Supplementary proteomics data file). Rather, the main difference was the decreased expression of anti-σ^70^ factors like MucAB, AlgQR, PP_3419, and PP_4364 (Fig. 2).

Considering that the growth rate in some conditions is faster for the (p)ppGpp^0^ strain compared to wild-type (Fig. 1d), despite clear differences in lag-times and final OD reached (Fig. S2B) and growth limitations visible on solid media (Fig. 1c), we hypothesize that the (p)ppGpp^0^ strain is unable to switch its metabolism to an energy-saving state as efficiently as wild-type. During fast growth it depletes its energy reserves quickly, resulting in a growth stall and death of some cells, seen from the decrease in CFU-s during stationary phase growth (Fig. 1b). This hypothesis is further supported by the (p)ppGpp^0^ strains’ known inability to induce alternative stress sigma factors (Jishage et al. 2002), which can be compensated by the anti-σ^70^ factors (AlgQR/MucAB/FliA/PP_3419/PP_4364) (Dove and Hochschild 2001, Schofield et al. 2021), all of which are downregulated in the (p)ppGpp^0^ strain in minimal media (Fig. 2).

### (p)ppGpp^0^  *P. putida* PaW85 membrane is permeable in the stationary phase

**Figure 3.**
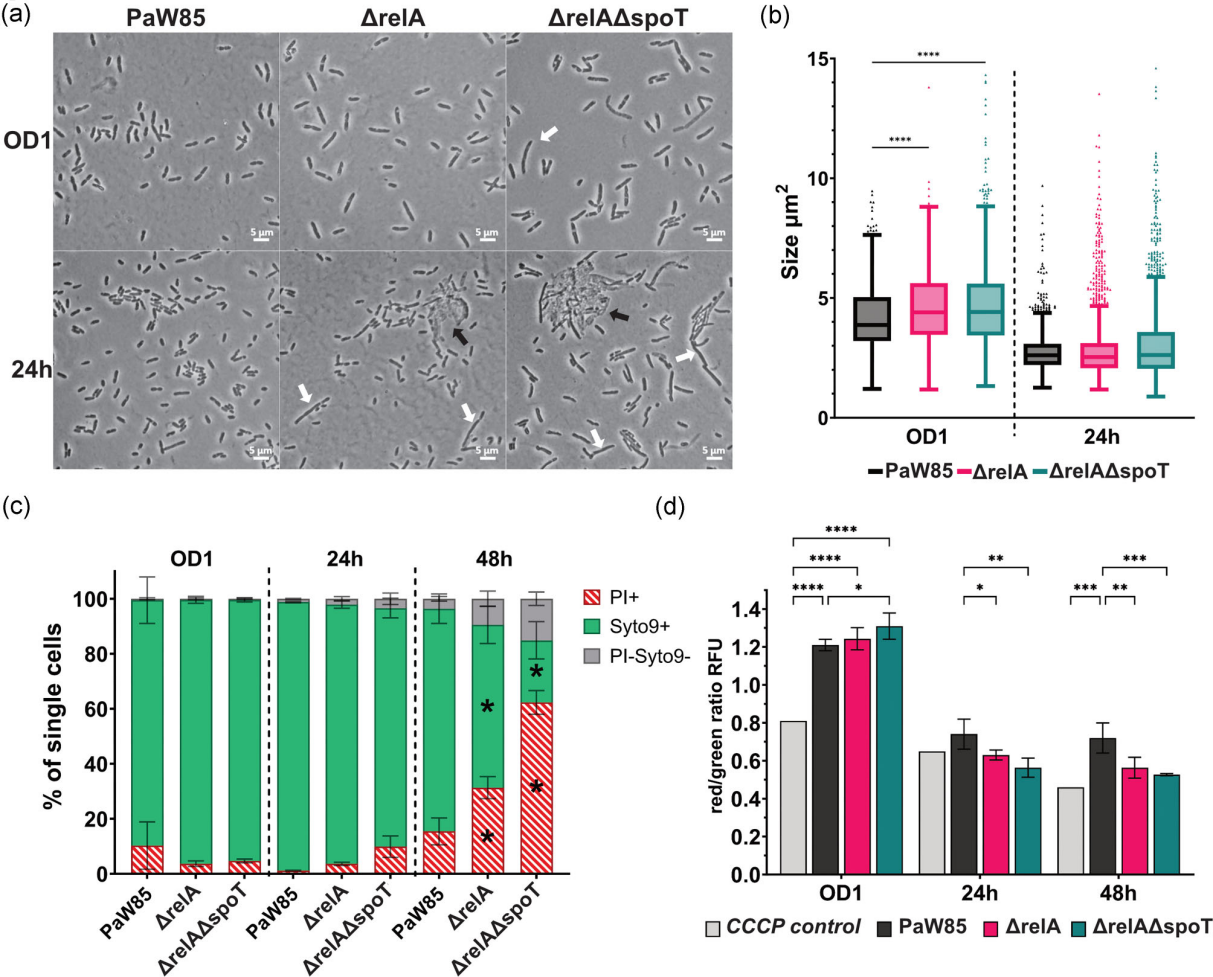
(p)ppGpp affects *P. putida* cell morphology and permeability in liquid LB medium. *Pseudomonas putida* PaW85 and its *relA* deletion (ΔrelA) and (p)ppGpp^0^ derivative (ΔrelAΔspoT) populations were compared in late exponential phase (OD1), stationary phase (24 h), and (in c and d) late stationary phase (48 h). (a) Microscopy images of LB-grown cultures. White arrows indicate elongated cells and black arrows the aggregates present in stationary phase (p)ppGpp-deficient cultures. (b) Box plots with Tukey whiskers and individual outliers of single cell sizes (area) analysed from microscopy images using MicrobeJ. Brown-Forsythe and Welch ANOVA with Dunnett’s T3 multiple comparisons test with individual variance. (c) Population differences from flow cytometry analysis: cells stained with propidium iodide (PI), Syto 9, and cells with reduced fluorescence intensity are brought out. Presented are means with SD (*N* = 3). Mutants were compared to wild-type in each time point with ordinary two-way ANOVA with Tukey’s multiple comparisons test with single pooled variance. (d) Flow cytometry analysis of cells stained with membrane potential indicator dye DiOC_2_(3). Geometric means of red and green fluorescence of the single cell population were analyzed. Higher red/green ratio indicates higher membrane potential. Means ± SD (*N* = 3). Ordinary two-way ANOVA with uncorrected Fisher’s Least Significant Difference (LSD) test with a single pooled variance. *P-value <.05; **P-value <.005; ***P-value < .001; ****P-value <.0001; RFU—relative fluorescence unit.

Our experiments with the (p)ppGpp^0^ strain showed that although the density of the bacterial cultures was similar or even higher for the (p)ppGpp^0^ strain, the colony counts were lower (Fig. 1b). Observing the cells under the microscope suggested that the (p)ppGpp^0^ bacteria are slightly larger compared to the wild-type (Fig. 3a and b) in the late exponential phase (OD 1) and form elongated cells and aggregates in the stationary phase (Fig. 3a). Although the average size of cells in 24 h cultures remains unchanged, the variability and the extreme values increased remarkably for both ΔrelA and ΔrelAΔspoT strains (compare 1^st^ and 3^rd^ quartiles and maximum values in Table S3, Fig. 3b). To know if just the observed elongated cells caused the increase in OD and decrease in the CFU measurements, or something else was happening to the population of (p)ppGpp^0^ strain, we carried out flow cytometry experiments for late exponential phase (OD_580_ 1), stationary phase (growth for 24 h) and late stationary phase cells (growth for 48 h). Staining the cells with LIVE/DEAD™ BacLight™ Bacterial Viability Kit (Thermo Fisher Scientific) showed a formation of several different subpopulations depending on the entrance of the two DNA stains (Fig. S1). Moreover, we observed that in the stationary phase, the amount of PI-permeable cells increases in (p)ppGpp absence, and a fraction of dead cells (lowered fluorescence intensity, decreased DNA content) starts to appear (Fig. 3c and for the initial number of cells for the calculation of subpopulation ratios, Fig. S4), suggesting severe membrane defects of the cells. Moreover, in the late stationary phase, the fraction of cells with PI-permeable membrane increased to over 60% for the (p)ppGpp^0^ population, and a fraction of dead cells increased to over 15%, compared to around 10% and <5% for the wild-type population, respectively (Fig. 3c). In the late stationary phase, the membrane defects also become visible for ΔrelA strain, although on a much lesser scale than for ΔrelAΔspoT ((p)ppGpp^0^).

To see whether membrane polarization is harmed in the (p)ppGpp^0^ strain, we stained the cells with DiOC_2_(3), which switches its red fluorescence to green in case of depolarization, and DiSC_3_(5), that stains only cells with depolarized membrane. Analysis of exponential phase cells showed no significant difference between the strains, yet in stationary phase, the ratio of red fluorescent cells started to increase in (p)ppGpp^0^ population, whereas in the late stationary phase, there was significant difference in the fluorescence ratio analysed with DiOC_2_(3) (Fig. 3d). This result was further confirmed by staining with DiSC_3_(5) that showed an increased subpopulation of cells with depolarized membrane in late stationary phase for the (p)ppGpp^0^ strain compared to wild-type (Fig. S5).

A phenotype of elongated cells of *E. coli relA spoT* double mutant was observed already in 1991 when growing cells on minimal media supplemented with all amino acids (Xiao et al. 1991). This morphology possibly originates from (p)ppGpp involvement in cell division (Magnusson et al. 2005). Nevertheless, for *E. coli*, the morphology is restored to a normal level on LB media (Xiao et al. 1991), whereas we visualized the aggregates and elongated cells for *P. putida* grown in LB rich media (Fig. 2a).

Studying the populations with flow cytometry, we observed that the lack of stringent response leads to an impaired membrane integrity during growth in the stationary phase (Figs. 3c and S4) demonstrated by the entrance of PI to the cells. In the late stationary phase, over half of the population of (p)ppGpp^0^ cells have a PI-permeable membrane, and a large subpopulation of cells have reduced DNA content, suggesting severe membrane defects. These defects could be the cause of the aggregates observed in microscopy imaging (Fig. 3a). Moreover, we see that (p)ppGpp^0^  *P. putida* might suffer from membrane depolarization, further supporting the hypothesis of energetic depletion of the strain.

**Figure 4.**
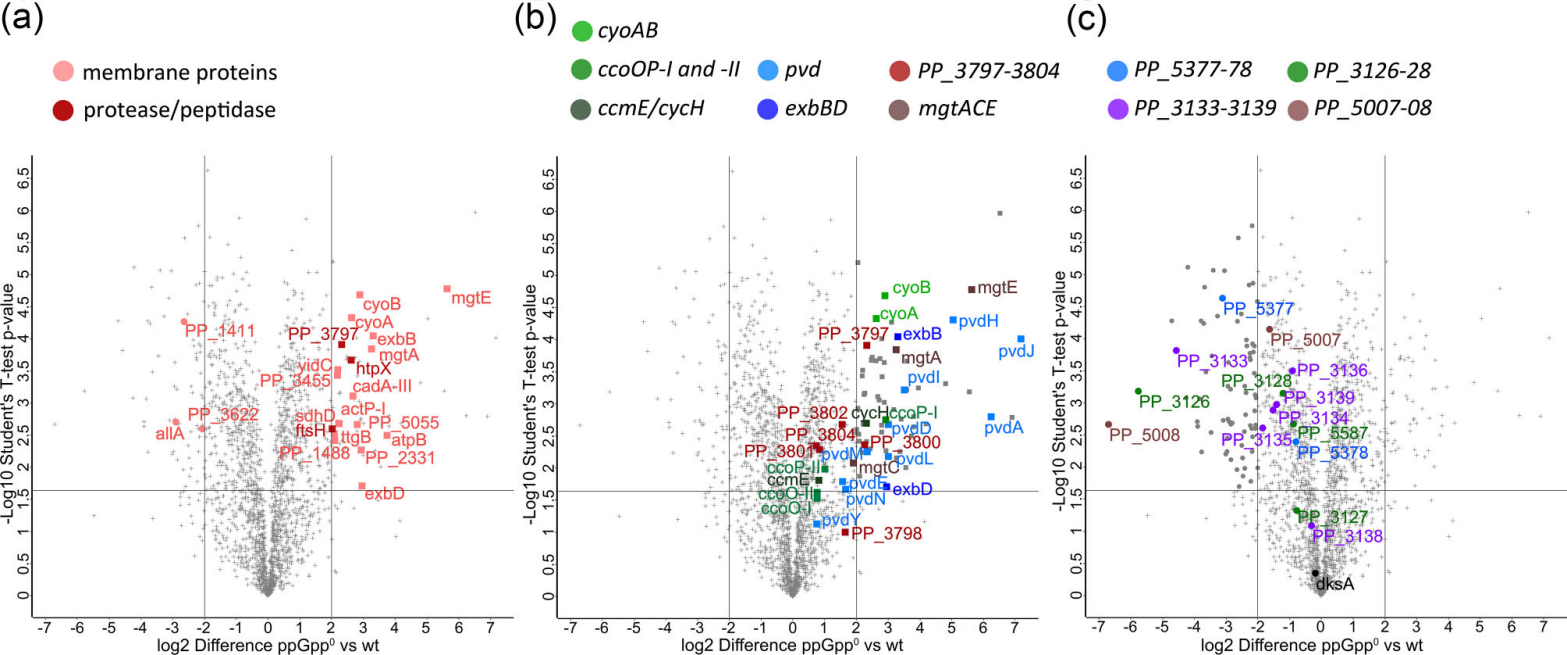
Differentially expressed genes in stationary phase (p)ppGpp^0^ PaW85 compared to wild-type grown in LB. Vertical lines show at least 4-times different expression, horizontal line marks the statistical significance of the difference (Student’s T-test; Benjamini-Hochberg multiple comparison test with false discovery rate 0.05). (a) Legend above shows the highlighted membrane protein groups: proteases, peptidases and all other membrane proteins both for decreased (circles) and increased (squares) expression. (b) Genes with significantly increased expression in (p)ppGpp absence (squares). Operons with clearly increased expression are brought out with colors shown in the figure legend above — cytochrome biosynthesis: cytochrome bo(3) (*cyoAB*) ; cbb-type cytochorome c (*ccoOP-I-II*); cytochrome c (*cycH, ccmE*); *mgtA, mgtC, mgtE* (Magnesium transport system); cation ABC transport operon (PP_3797–3804); and pyoverdine synthesis (*pvd*) and transport-powering genes (*exbBD*). (c) Genes with significantly decreased expression in (p)ppGpp absence (circles). Main operons with decreased expression in (p)ppGpp absence: polyhydroxyalkanoate synthesis (PP_5007–5008), polysaccharide biosynthesis/export (PP_3126–3128); Copper resistance genes (PP_5377–5378). DksA expression is similar in both strains (labelled in c). Data were analyzed using Perseus software.

We also compared the full proteome of stationary phase wild-type and (p)ppGpp^0^  *P. putida* in LB. We identified three at least four times downregulated and 18 at least four times upregulated membrane proteins comparing (p)ppGpp^0^ strain to wild-type (Fig. 4a). Among these were membrane proteases FtsH amd HtpX and peptidase PP_3797 (Winsor et al. 2016) that are probably involved in degradation of damaged or misfolded membrane proteins. This could lead to an imbalance of membrane proteins and result in a damaged membrane of (p)ppGpp^0^ cells. It has also been suggested for *Vibrio cholerae* that (p)ppGpp^0^ membrane integrity loss could result from deregulation of expression of cell wall or membrane proteins (Chawla et al. 2025) or lipopolysaccharides (LPSs) (Schäkermann et al. 2013) in the lack of (p)ppGpp.

Many of the other overexpressed genes identified in (p)ppGpp^0^ strain seem to be compensatory expressions as a result to membrane stress. For example, the *pvd* pyoverdine synthesis pathway important for siderophore production in *P. putida* and the ExbBD proteins are responsible for the synthesis and transport of pyoverdine siderophores. Both groups of genes are strongly overexpressed in *P. putida* (p)ppGpp^0^ strain. The upregulation of *pvd* genes has also been previously reported upon membrane stress of *P. putida* deficient in the ColRS two-component system (Mumm et al. 2016) and TonB–PocAB system (Ainsaar et al. 2019), suggesting that the increased Pvd-type siderophore production is rather a compensatory effect upon the loss of membrane integrity than a cause of it.

A few patterns were also visible for the downregulated genes. The expression of operons for energy saving/storing mechanisms were generally downregulated compared to wild-type. For example, polyhydroxyalkanoate synthesis (PP_5007–5008) (Winsor et al. 2016), polysaccharide synthesis/export (PP_3126–3128) (Winsor et al. 2016), and most of *pea* exopolysaccharide synthesis operon (PP_3132–3142 genes) (Nielsen et al. 2011). These data suggest that wild-type cells have switched to energy-saving mode, but the (p)ppGpp^0^ cells have not (Fig. 4c). Also, copper resistance genes PP_5377 and PP_5378 (Winsor et al. 2016) were downregulated in (p)ppGpp absence. The full list of genes significantly up- or downregulated in are available in the Supplementary proteomics data file.

The effect of the absence of (p)ppGpp on membrane integrity has also been observed for *V. cholerae*, demonstrated by an increased uptake of PI and NPN (1-N-phenylnaphthylamine) that allow the detection of compromised inner and outer membrane, respectively (Chawla et al. 2025). The morphology of (p)ppGpp^0^  *E. coli* is also elongated and aggregating (Magnusson et al. 2007, Das et al. 2009), and the expression of DksA alleviates the morphology defects (Magnusson et al. 2007), but not other (p)ppGpp^0^ phenotypes (Brown et al. 2002). DksA is a transcriptional regulator tightly interacting with (p)ppGpp (Brown et al. 2002, Gourse et al. 2018). The deletion of *dksA* causes similar effects as (p)ppGpp^0^ strains, including inability to grow on minimal media for *E. coli* (Brown et al. 2002) and outer membrane defects for *V. cholerae* (Chawla et al. 2025). Although the membrane and cell morphology effects caused by the lack of (p)ppGpp seem to be interconnected with the DksA protein in different bacteria, the exact molecular background of the changes in membrane integrity have not been determined to our knowledge for any of the bacterial species. Notably, the amount of DksA protein was the same in LB-grown *P. putida* (p)ppGpp^0^ and wild-type (Fig. 4c).

These data together suggest that although the (p)ppGpp^0^ derivative of *P. putida* PaW85 can maintain its growth and physiology in exponential growth, it loses membrane integrity upon switching to stationary phase suggesting an inability to switch to energy-saving mode. That probably also creates imbalance of membrane proteins that leads to depolarized membrane of the (p)ppGpp^0^  *P. putida*.

### *P. putida* PaW85 (p)ppGpp^0^ strain tolerates chemical stress well

**Figure 5.**
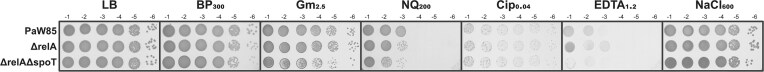
Growth of *P. putida* PaW85 wild-type, ΔrelA, and (p)ppGpp^0^ (ΔrelAΔspoT) cells on solid LB media with different stress-causing chemicals: benzylpenicillin (Bp, cell wall damage), gentamycin (Gm, translation inhibition), nitroquinoline (NQ, DNA damage), ciprofloxacin (Cip, DNA damage), ethylenediaminetetraacetic acid (EDTA, membrane damage), and NaCl (osmotic stress). Number indicates concentration µg/ml (Bp, Gm, Cip) or mM (NQ, EDTA, NaCl). Representative images from at least three biological replicates are presented. Plates were incubated at 30°C for 24 h or (48 h for EDTA). Dilution series have been straightened digitally for visualization purposes.

To assess the chemical stress tolerance of the (p)ppGpp^0^ strain, we observed growth of *P. putida* PaW85 and its ΔrelA and ΔrelAΔspoT (ppGpp^0^) derivatives on LB medium containing different chemicals and antibiotics that induce different types of stresses to bacteria: benzylpenicillin (Bp), Gentamycin (Gm), Nitroquinoline (NQ), Ciprofloxacin (Cip), Ethylenediaminetetraacetic acid (EDTA), and NaCl (types of stresses together with MIC values are brought in Table S4). As expected, (p)ppGpp^0^ bacteria tolerate stress conditions slightly less than the wild-type *P. putida* (Fig. 5). Surprisingly, in the presence of EDTA, agent disrupting the cell membrane (Marvin et al. 1989), the *spoT* bacteria without RelA can grow better than the wild type. Nevertheless, when no (p)ppGpp can be produced, the cells tolerate EDTA even less than the wild-type strain. Also, we did not observe any drastic changes between wild type and (p)ppGpp^0^ strain in the MIC values for any of the tested chemicals (Table S3). To confirm our results, we also followed the growth of the wild-type and (p)ppGpp^0^ strain in liquid LB containing three of the tested chemicals (Bp, Cip, and EDTA; Fig. S6), obtaining similar results to the solid media assay.

Regardless of the observed clear membrane defect of the bacteria (Fig. 3), the stress survival has changed surprisingly little, both on solid media (Fig. 5) and in liquid cultures (Fig. S6, Table S4). These data show that the growth upon different stresses is generally slightly reduced when (p)ppGpp is not produced in the cells, whereas the overall MIC values stay stable regardless of the reduction or absence of cellular (p)ppGpp levels.

We conclude that the tolerance to the chemical stressors is only slightly reduced by the lack of (p)ppGpp, whereas the resistance (MIC) remains unchanged for the (p)ppGpp^0^ strain for all the tested chemicals. Although previous data is in agreement with our results showing that the MIC values of (p)ppGpp^0^  *E. coli* remain unchanged for aminoglycoside gentamycin (Gm) and fluoroquinolone ofloxacin (Grucela and Zhang 2023), compared with Gm and ciprofloxacin (Cip) for *P. putida* (Table S4), our results are in contrast to earlier reports on resistance to β-lactam antibiotics. The resistance of *E. coli* to ampicillin decreases upon lack of (p)ppGpp (Grucela and Zhang 2023), whereas we observed only a slight growth reduction and no difference in MIC when testing benzylpenicillin (Fig. 5, Table S3 and Fig. S6). The two antibiotics have a similar function and differ only by an added amino group to the penicillin core for ampicillin (Raynor 1997). This means that the (p)ppGpp effects on chemical stresses are different for these bacterial species.

### Lack of (p)ppGpp has opposing effects on phage infection

As chemical stressors did not show a strong effect on (p)ppGpp^0^  *P. putida*, we decided to determine how the stringent response could be involved in a different stressful situation—phage infection. For that, we compared the phage infection efficiency for cells with wild-type (p)ppGpp levels and the (p)ppGpp^0^ strain. We screened through the CEPEST collection, creating a bacterial lawn with bacteria from the late stationary phase. As some of the phages (e.g. the G3 phage genus cluster) are strongly inhibited by prophages present in *P. putida* PaW85 genome (Brauer et al. 2024), we created a (p)ppGpp^0^ strain in a prophage-free background of *P. putida* PaW85 (Δ4ΦΔrelAΔspoT) to screen the effect of (p)ppGpp absence for the phages of genus clusters G3 and G9, and phages Kompost-1 (G5) and Kompost-2 (G7) (Brauer et al. 2024).

**Figure 6.**
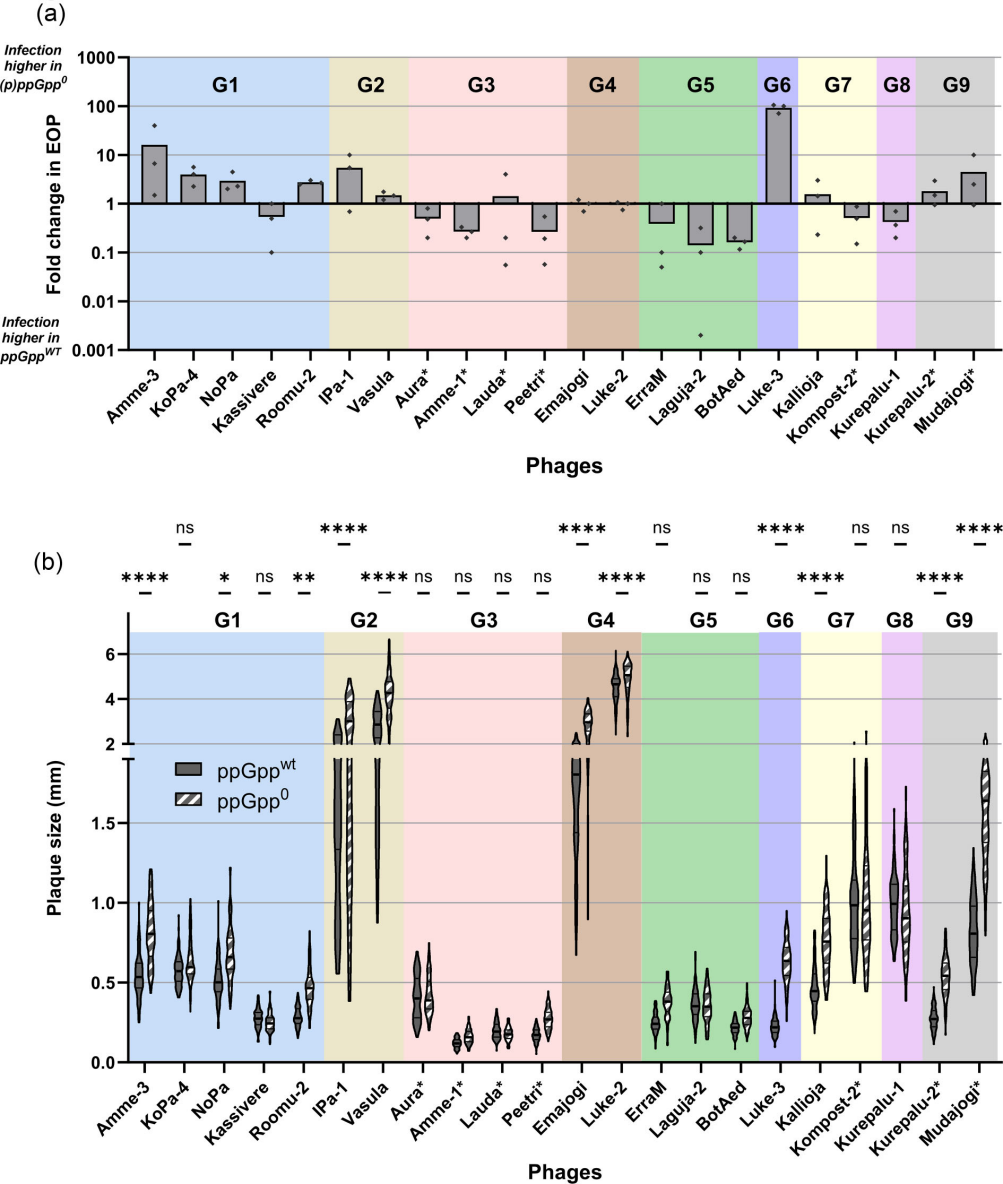
Phage infection depends on the stringent response. (a) Fold change in the EOP of representative phages of each CEPEST phage species (ppGpp^0^/ppGpp^wt^), bars represent average values, and dots the three independent experiment results. (b) Violin plot of phage plaque size on ppGpp^wt^ (grey filled) or ppGpp^0^ (grey striped) bacterial lawn. RM one-way ANOVA with Bonferroni’s multiple comparison with a single pooled variance test: *P-value <.05; **P-value <.005; ****P-value <.0001; ns—nonsignificant. The colors and G-numbers mark different CEPEST genus clusters, and an asterisk behind the phage name marks the phages tested in a prophage-free background.

By comparing the efficiency of plaquing (EOP) of CEPEST phages on the lawn of bacteria with functional stringent response ((p)ppGpp^wt^) to bacteria lacking (p)ppGpp production ((p)ppGpp^0^), we observed a variety of (p)ppGpp dependence in infection efficiency: from a 100-fold increase (Luke-3) to a 10-fold decrease (e.g. for Laguja-2 and BotAed) of infection of (p)ppGpp^0^ strain (Figs. 6a and S7). As the plaque-forming unit (PFU) counts reflect only the ability of phages to infect cells, rather than the infection process, we also measured the sizes of plaques for the representative species of the CEPEST collection and saw that the plaque sizes were often enlarged upon lack of (p)ppGpp in bacteria (Figs. 6b and S7A). Luke-3 phage has the highest infection efficiency difference as well as the largest increase of plaque sizes in the absence of (p)ppGpp (Figs. 6b and S7A). Markedly, the G4 phages (Emajogi and Luke-2), that showed no effect of EOP between the strains, have significantly larger plaques on the bacterial lawn of the (p)ppGpp^0^ strain, showing that the infection efficiency, but not the ability, depends on (p)ppGpp presence. Phages that had a decreased infection efficiency for (p)ppGpp^0^ strain showed no significant changes in the average plaque size (Kassivere, G3 and G5 phages, Fig. 6b).

Almost half of the representatives of CEPEST phage species infected the bacteria without (p)ppGpp more efficiently than the wild-type, having higher PFU counts and/or (in case of G4 phages, Emajogi and Luke-2) significantly larger plaque size on the lawn of (p)ppGpp^0^ bacteria (Figs. 6, and S7). As the stringent response is known to reduce translation, transcription, and replication efficiency upon stress (Potrykus and Cashel 2008, Srivatsan and Wang 2008, Dalebroux and Swanson 2012), the inability of the bacteria to do so in case of a phage attack may lead to higher infection rates for phages due to higher activity of biosynthesis pathways. Even if the infection ability did not differ according to the PFU counts (Emajogi and Luke-2), the plaques produced on (p)ppGpp^0^ bacterial lawn were, on average, bigger. Larger plaque sizes could correlate with both a larger burst size (Abedon and Yin 2009) or a shorter latent period (Abedon et al. 2003), so it may reflect a relaxed (uninhibited) replication and translation in (p)ppGpp absence.

However, nearly half of the species and over half of all the phages (most of G3, G5, and G8 genus cluster phages; Figs. 6 and S7) infected the (p)ppGpp^0^ strain less efficiently than the wild-type, suggesting that the lack of stringent response somehow protects the cells from infection, or that phages need stringent response to carry out their infection in most efficient level. Somewhat similar to what we observe with G3, G5, and G8 phages of CEPEST collection, has been previously reported for *P. aeruginosa* jumbo phage Paride, which also shows poorer infection for the (p)ppGpp^0^ strain compared to wild-type (Maffei et al. 2024). Paride phage can also proliferate in late stationary phase cultures, where cells are dormant (Maffei et al. 2024). Although there is little known about the mechanisms for the observed effects for the Paride phage, it could be that some phages have evolved to counteract bacterial stress response systems and started to require their presence for more efficient infection. The *Campylobacter jejuni* T4-like phage NCTC12673 seems to require functional oxidative stress processes to infect its host (Sacher et al. 2018). Thus, it appears that the networks between different host stress responses and phage infections are multifaceted and require further studies for elucidation.

### The changes in infection efficiency are not caused by adsorption rate difference

As another possible reason for the decreased or increased infection of (p)ppGpp^0^ bacteria, we also considered that the impaired membrane of (p)ppGpp^0^  *P. putida* may cause changes in phage receptors that can result in altered infection of phages (Egido et al. 2022) by inhibiting the first step of infection—adsorption. To test this hypothesis, we chose two phages that showed opposite effects in phage infection assays: Amme 3–presence of (p)ppGpp protects from phage infection (10-fold increase of infection of (p)ppGpp^0^ strain compared to wild-type) and Aura—presence of (p)ppGpp sensitizes the cells to phage infection (2–5-fold decrease of infection upon lack of (p)ppGpp). First, as adsorption assays are carried out in liquid cultures, we confirmed that the effects of the infection in liquid media correspond to the ones visualized on the solid medium. We saw that this indeed is the case, with the phage infection being around 10 times more (for Amme-3) or less (for Aura) efficient for the (p)ppGpp^0^ strain compared to the wild-type *P. putida* (Fig. S8).

**Figure 7.**
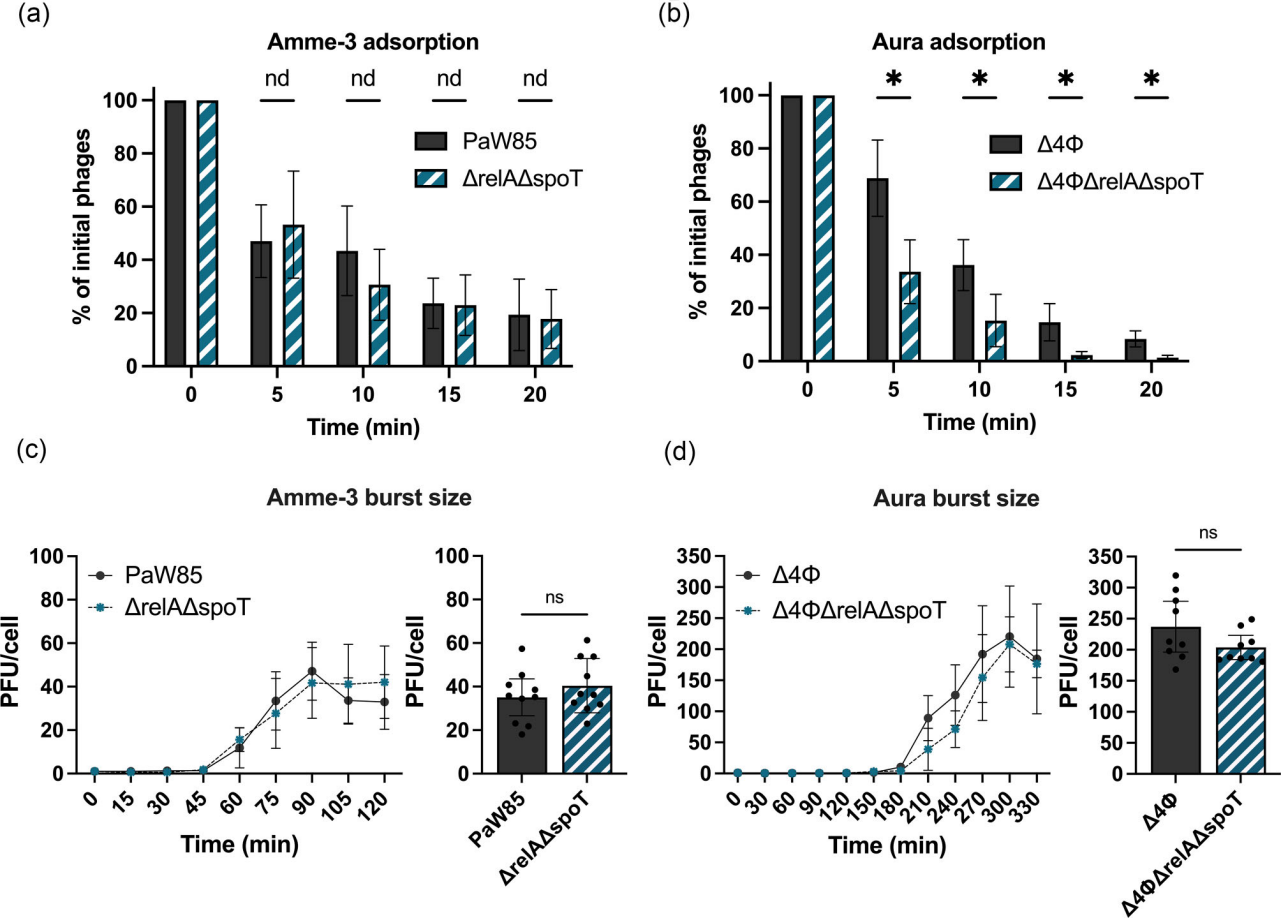
The stringent response effect on the infection of Amme-3 and Aura phages. (a) Infectious Amme-3 phages remaining in the supernatant after adsorption to *P. putida* PaW85 (filled dark grey) or its (p)ppGpp^0^ derivative (ΔrelAΔspoT, striped teal). (b) Infectious Aura phages remaining in the supernatant after adsorption to prophage-free *P. putida* PaW85 (Δ4ϕ, filled dark grey) or its (p)ppGpp^0^ derivative (Δ4ϕΔrelAΔspoT, striped teal). (c) The one-step Amme-3 phage growth curve developed under the MOI 0.1 for *P. putida* PaW85 (dark grey) and its (p)ppGpp^0^-derivative (ΔrelAΔspoT, teal). (d) The one-step Aura phage growth curve developed under the MOI 0.1 for prophage-free *P. putida* wild-type (Δ4ϕ, dark grey) and its (p)ppGpp^0^ derivative Δ4ϕΔrelAΔspoT (teal). Means ± 95% confidence intervals (*N* ≥ 3) are shown. (a) and (b) data were analysed using multiple unpaired t-test with a two-stage step-up method of Benjamini, Krieger, and Yekuteli; *—discovery; nd—non-discovery. (c) and (d) data were analysed using multiple unpaired t-test method of Mann–Whitney; ns—non-significant.

The adsorption of Amme-3 phage was surprisingly inefficient, with the unattached phages remaining stable at 20% of the initial amount by 15 and 20 min for both strains. Even more phages remained unattached when infecting prophage-free strain (Δ4ϕ) and its (p)ppGpp^0^ derivative (Δ4ϕΔrelAΔspoT) (Fig. S9). The adsorption on the (p)ppGpp^0^ strains was not significantly changed in either case, with the average ratio of unbound phages for both of the tested strains staying similar (Figs. 7a and S9). These results clearly show that (p)ppGpp provides protection after the phage adsorption to the cell, and the more efficient infection of (p)ppGpp^0^ strain does not result from better adsorption due to higher membrane permeability of the stringent response mutant.

For Aura phage, we see a much higher rate and level of adsorption for the (p)ppGpp^0^ cells (prophage-free background, Δ4ϕΔrelAΔspoT, Fig. 7b). The phages adsorb to the cells faster, and the level of unattached phages remains higher for bacteria with normal (p)ppGpp production (Δ4ϕ) after 20 min, with 90%–95% of phages having attached to the Δ4ϕ strain compared to around 99% on its (p)ppGpp^0^ derivative (Fig. 7b). Thus, the changes in membrane integrity in (p)ppGpp absence seem to lead to a more efficient adsorption of the Aura phage. This, in turn, shows that the infection process for Aura inside the cells must be strongly affected by (p)ppGpp, as the (p)ppGpp^0^ cells are infected less efficiently both in liquid and solid media.

Our hypothesis that the membrane defects of the (p)ppGpp^0^ strain reduce infection due to lowered adsorption efficiency seems not to be true (at least for G3 phages, as shown for a representative G3A phage Aura), as the adsorption of Aura was increased (not decreased) for (p)ppGpp^0^ cells. The increased adsorption efficiency could be due to better access to the binding receptor of Aura on the cells upon the lack of (p)ppGpp. Aura phage is LPS-dependent (Brauer et al. 2024) and in *E. coli* (p)ppGpp regulates LPS biosynthesis, with the lack of (p)ppGpp leading to the deregulation of the degradation of LpxC (Schäkermann et al. 2013), one of the first key enzymes in the lipid A synthesis pathway (Young et al. 1995, Sorensen et al. 1996, Zhou and Zhao 2017). Upon slow growth (p)ppGpp^0^  *E. coli* cells degrade Lpx more slowly, causing its accumulation and leading to elongated cells. We also observe elongated cells upon deletion of *relA* and *spoT* genes in the stationary phase (Fig. 3a), suggesting that a similar deregulation of lipid A synthesis could also happen in *P. putida*. This, in turn, could potentially mean better access or higher expression of Aura phage receptor on the cell surface and offer an explanation to the increased adsorption of Aura to (p)ppGpp^0^ cells.

Similarly to Aura phage, Amme-3 infection is dependent on LPS, as it was unable to infect cells lacking the WbpL (Brauer et al. 2024), a glycosyltransferase required for the synthesis of O-antigen (Rocchetta et al. 1998). Nevertheless, contrary to Aura phage, the adsorption of Amme-3 was not dependent on cellular (p)ppGpp levels (Fig. 5a). Although both Amme-3 and Aura infections are LPS-dependent, the exact receptor for either of the phages is yet unknown. The difference in the (p)ppGpp effect on the adsorption of the Amme-3 and Aura phages suggests that although the infection of both phages requires O-antigen on the LPS, the precise receptor they recognize is probably different. The one for Amme-3 remains unchanged in (p)ppGpp absence, whereas the one of Aura is either overproduced or overexposed in the absence of cellular (p)ppGpp.

### Different phage infection steps are altered due to absence of cellular (p)ppGpp

As the infection efficiency changes could not be explained by phage adsorption, we decided to study the whole infection cycle of the phages Aura and Amme-3. For Aura phage, we observed a very long infection cycle, with the burst starting after 3 or 3.5 h after adsorption. The latent period of infection was increased in the absence of (p)ppGpp by around 30 min. The burst size of the phages infecting the two strains was not significantly different (Fig. 7). Thus, the only anomaly of Aura phage infection of (p)ppGpp^0^ strain is the prolonged latent period. The slower production of phages may explain the lowered infection efficiency observed in the infection assay in liquid media, but could also offer an explanation to the lower PFU counts. The infection assays on solid media were carried out by using stationary phase cells, and as (p)ppGpp is more important for the cells in the stationary phase (Figs. 1 and 3), it could be that the delay in burst is even more pronounced in that assay. Thus, *P. putida*, having an average doubling time at 20°C of 150 min (Tamman et al. 2014), might be able to overgrow the phage plaques, inhibiting the visualization of infection.

**Figure 8.**
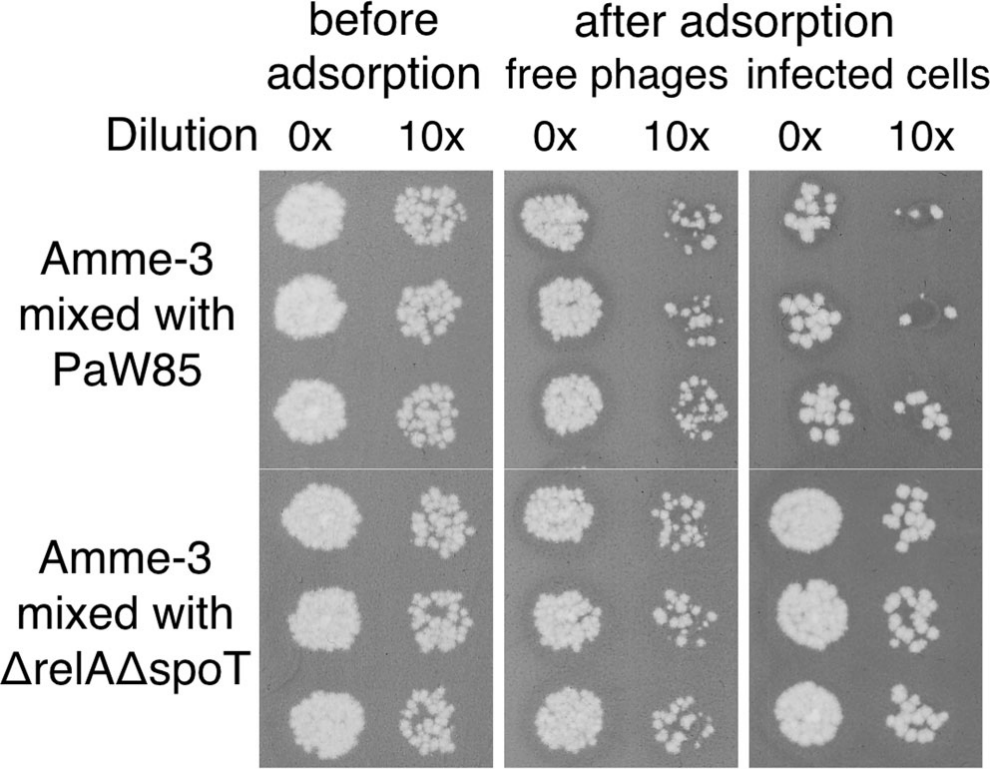
Amme-3 phages cannot efficiently infect all wild-type PaW85 cells. Phage plaques on bacterial lawn after mixing Amme-3 phage with either *P. putida* PaW85 or its (p)ppGpp^0^ derivative (ΔrelAΔspoT) at MOI of 0.1. First panel shows amount of phages in initial mix, second one phages remaining in the supernatant after 20 min adsorption and the third—phage plaques created by infected cells just after 20 min of adsorption.

The latent period of the Amme-3 phage does not depend on the presence of (p)ppGpp (Fig. 7). Also, the burst is much faster, starting already after 45 min after the adsorption step has finished. Nevertheless, as observed for Aura, also the burst size of the Amme-3 phage is lower in (p)ppGpp^0^ background, conflicting with the plaque size measurements that showed a significantly increased plaque size in (p)ppGpp absence. Surprisingly, although adsorption of the Amme-3 phage showed no dependence on (p)ppGpp in the presence or absence of prophages (Figs. 7 and S9). At the starting point of burst size experiments, the number of phages differed 10 times (compare PaW85 and its (p)ppGpp^0^ derivative ΔrelAΔspoT on Fig. 8), suggesting that not all adsorbed phages can carry out productive infection in the cell and thus not all infected cells result in plaque formation from the wild-type cells.

**Figure 9.**
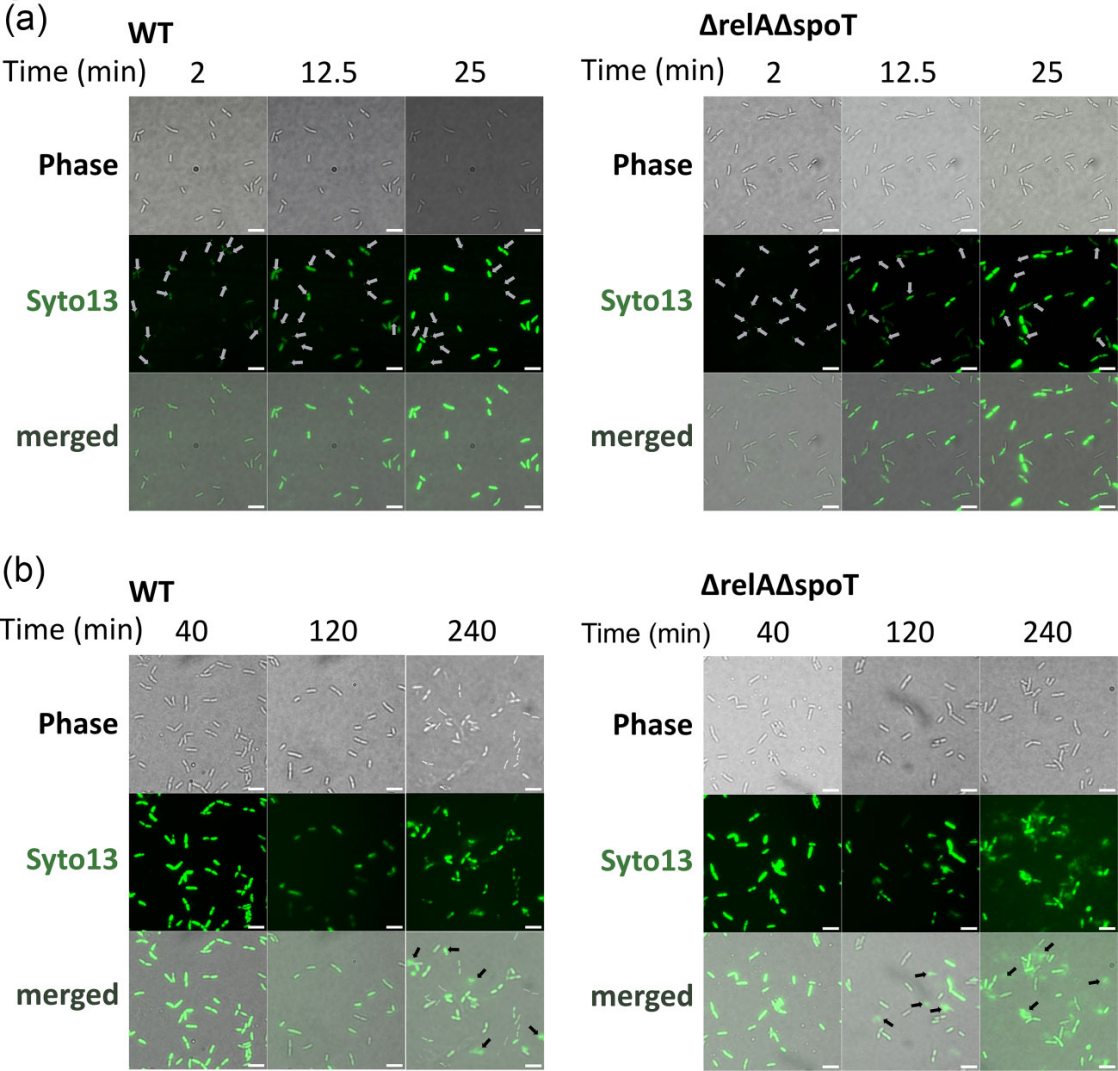
Microscopy imaging of the stringent response effect on Amme-3 phage infection of *P. putida* PaW85 wild-type cells (on the left) or its (p)ppGpp^0^ derivative (ppGpp^0^–on the right). (a) Time-lapse of Amme-3 phage binding to and infecting cells imaged on glass. Amme-3 phage particles stained with Syto 13 appear as green spots (marked with grey arrows). Successful infection is visible as the infected bacteria turn green over time. (b) *P. putida* cells infected with Syto 13-stained Amme-3 phage—stages after adsorption imaged on agarose pads. Cell-free Syto 13 dye, probably freed from lysed cells, is marked with black arrows. Scale bars on images represent a 10 μm length.

To check which step of infection is inhibited for the Amme-3 when infecting the wild-type *P. putida*, we stained the Amme-3 phage with Syto 13 and observed the injection and lysis of the phages to either *P. putida* PaW85 or its (p)ppGpp^0^ derivative. We saw that cells of both strains gradually turn green when infected with Syto 13 colored phages in 25 min (Fig. 9a, Supplementary videos 1 and 2), but the lysis occurs much more in the (p)ppGpp^0^ background (Fig. 9b), demonstrating that even though the wild-type *P. putida* cells turn green, the lysis seems not to follow for many of the cells. This means that (p)ppGpp in wild-type *P. putida* PaW85 acts as a phage defence determinant resulting in unproductive (or strongly prolonged) infection of *P. putida* cells.

## Concluding remarks

In conclusion, with this work, we showed that the (p)ppGpp^0^ strain of *P. putida* PaW85 is prototrophic and has slightly lowered stress tolerance. In the stationary phase, the cells have problems with membrane integrity, determined by an increased subpopulation of PI-permeable and dead cells. Moreover, it seems that (p)ppGpp-deficient strain suffers from a slightly depolarized membrane. The strain experiences opposite effects upon phage infection. Surprisingly, although a major stress response is missing in these bacteria, not all phages can infect the bacteria better. On the contrary, around half of all the tested phages show an unchanged or decreased infection efficiency in the absence of the stringent response. We show that even if the membrane defects may increase the adsorption of the phage Aura, it does not explain the decreased infection efficiencies observed, and we can only conclude that the phage infection cycle of Aura must be strongly influenced by (p)ppGpp to show an opposite effect on infection efficiency compared to adsorption. Indeed, the latent period of Aura infection is longer in (p)ppGpp absence, which may lead to the observed lowered infection efficiency as it takes longer for the new progeny of phages to be released. For Amme-3 phages neither the adsorption nor infection cycle itself is changed, rather the (p)ppGpp levels in some wild-type cells seem to provide a strong protection against Amme-3 phages that results in an unproductive infection of wild-type bacteria. Thus, we can conclude that (p)ppGpp can affect several steps in phage infection cycle in both positive and negative manner, depending on the phage.

Overall, (p)ppGpp seems to be less important for the survival of *P. putida* in stress conditions than in other bacterial species, but still causes global changes in gene regulation and bacterial energetic states, affecting downstream processes, such as membrane integrity and phage infection cycles.

## Supplementary Material

uqaf048_Supplemental_Files
