# Supplementary material for: The opposite effects of stringent response on phage infection of Pseudomonas putida
Source: Microlife. 2026 Jan 2;7:uqaf048. doi: 10.1093/femsml/uqaf048 (PMC12814882; doi:10.1093/femsml/uqaf048)
Supplement: uqaf048_Supplemental_Files [file uqaf048_supplemental_files.zip › Supplementary File 1 Tables_REVISED.pdf]

Table S1 Strains, plasmids and bacteriophages used in this study

Strain /plasmid/phage	Description	Source
***E. coli* strain**		
Dh5α λpir	λpir lysogen of DH5α	(Martínez-García and de Lorenzo 2011)
***P. putida* strains**		
PaW85	wild type, isogenic to KT2440	(Bayley *et al.* 1977)
Δ4ϕ	PaW85 with deletion of four prophages	(Brauer *et al.* 2024)
ΔrelA	PaW85 with deleted *relA*	This study
Δ4ϕΔrelA	Δ4ϕ with deleted *relA*	This study
ΔrelAΔspoT (ppGpp^0**)**	ΔrelA with deleted *spoT*	This study
Δ4ϕΔrelAΔspoT (Δ4ϕppGpp^0)	Δ4ϕΔrelA with deleted *spoT*	This study
**Plasmids**		
pEMG	Plasmid for homologous recombination, lacZα with two flanking I-SceI sites (Km^r)	(Martínez-García and de Lorenzo 2011)
pSW(I-SceI)	Plasmid coding for I-SceI endonuclease for allelic exchange experiments (Bp^r)	(Wong and Mekalanos 2000)
pEMG-ΔrelA	pEMG containing a chimeric DNA fragment for deleting *relA*	This study
pEMG-ΔspoT	pEMG containing a chimeric DNA fragment for deleting *spoT*	This study
**Phages (CEPEST collection)**	Genus and species cluster	
**Amme-3**	G1 genus , species cluster A	(Brauer *et al.* 2024)
**KoPa-4**	G1 genus , species cluster B	(Brauer *et al.* 2024)
**NoPa**	G1 genus , species cluster C	(Brauer *et al.* 2024)
**Kassivere**	G1 genus , species cluster D	(Brauer *et al.* 2024)
**Roomu-2**	G1 genus , species cluster E	(Brauer *et al.* 2024)
**IPa-1**	G2 genus , species cluster A, representative phage	(Brauer *et al.* 2024)
AnF1	G2 genus , species cluster A	(Brauer *et al.* 2024)
Eino	G2 genus , species cluster A	(Brauer *et al.* 2024)
Haage	G2 genus , species cluster A	(Brauer *et al.* 2024)
Hammaste-5	G2 genus , species cluster A	(Brauer *et al.* 2024)
Issaku	G2 genus , species cluster A	(Brauer *et al.* 2024)
Kanksi	G2 genus , species cluster A	(Brauer *et al.* 2024)
Keeri	G2 genus , species cluster A	(Brauer *et al.* 2024)
Keila	G2 genus , species cluster A	(Brauer *et al.* 2024)
KoPa-1	G2 genus , species cluster A	(Brauer *et al.* 2024)
Kriimani	G2 genus , species cluster A	(Brauer *et al.* 2024)
Maksa	G2 genus , species cluster A	(Brauer *et al.* 2024)
Nouni	G2 genus , species cluster A	(Brauer *et al.* 2024)
Roomu-1	G2 genus , species cluster A	(Brauer *et al.* 2024)
Torvandi	G2 genus , species cluster A	(Brauer *et al.* 2024)
Torve	G2 genus , species cluster A	(Brauer *et al.* 2024)
Voja-1	G2 genus , species cluster A	(Brauer *et al.* 2024)
**Vasula**	G2 genus , species cluster B, representative phage	(Brauer *et al.* 2024)
**Aura**	G3 genus , species cluster A, representative phage, tested with Δ4ϕ	(Brauer *et al.* 2024)
Illi-2	G3 genus , species cluster A, tested with Δ4ϕ	(Brauer *et al.* 2024)
SKa-4	G3 genus , species cluster A, tested with Δ4ϕ	(Brauer *et al.* 2024)
**Amme-1**	G3 genus , species cluster B, representative phage, tested with Δ4ϕ	(Brauer *et al.* 2024)
KoPa-5	G3 genus , species cluster B, tested with Δ4ϕ	(Brauer *et al.* 2024)
Pori-4	G3 genus , species cluster B, tested with Δ4ϕ	(Brauer *et al.* 2024)
Voja-6	G3 genus , species cluster B, tested with Δ4ϕ	(Brauer *et al.* 2024)
**Lauda**	G3 genus , species cluster C, tested with Δ4ϕ	(Brauer *et al.* 2024)
**Peetri**	G3 genus , species cluster D, tested with Δ4ϕ	(Brauer *et al.* 2024)

**Emajogi**	G4 genus , species cluster A	(Brauer *et al.* 2024)
**Luke-2**	G4 genus , species cluster B	(Brauer *et al.* 2024)
**ErraM**	G5 genus , species cluster A, representative phage	(Brauer *et al.* 2024)
ErraS	G5 genus , species cluster A	(Brauer *et al.* 2024)
Vanda	G5 genus , species cluster A	(Brauer *et al.* 2024)
**Laguja-2**	G5 genus , species cluster B, representative phage	(Brauer *et al.* 2024)
Hammaste-2	G5 genus , species cluster B	(Brauer *et al.* 2024)
Ihaste	G5 genus , species cluster B	(Brauer *et al.* 2024)
Illi-1	G5 genus , species cluster B	(Brauer *et al.* 2024)
Kaagvere	G5 genus , species cluster B	(Brauer *et al.* 2024)
Kadastiku	G5 genus , species cluster B	(Brauer *et al.* 2024)
KiKu	G5 genus , species cluster B	(Brauer *et al.* 2024)
Konnatiik	G5 genus , species cluster B	(Brauer *et al.* 2024)
Kurenuki	G5 genus , species cluster B	(Brauer *et al.* 2024)
Laguja-5	G5 genus , species cluster B	(Brauer *et al.* 2024)
Luutsna-1	G5 genus , species cluster B	(Brauer *et al.* 2024)
Luutsna-3	G5 genus , species cluster B	(Brauer *et al.* 2024)
Luutsna-6	G5 genus , species cluster B	(Brauer *et al.* 2024)
Mora-3	G5 genus , species cluster B	(Brauer *et al.* 2024)
Mora-5	G5 genus , species cluster B	(Brauer *et al.* 2024)
Paidla	G5 genus , species cluster B	(Brauer *et al.* 2024)
Pori-2	G5 genus , species cluster B	(Brauer *et al.* 2024)
SaviPeeda	G5 genus , species cluster B	(Brauer *et al.* 2024)
Villemi	G5 genus , species cluster B	(Brauer *et al.* 2024)
Kompost-1	G5 genus species cluster B*	(Brauer *et al.* 2024)
SKa-3	G5 genus species cluster B*	(Brauer *et al.* 2024)
**BotAed**	G5 genus , species cluster C	(Brauer *et al.* 2024)
**Luke-3**	G6 genus , species cluster A, representative phage	(Brauer *et al.* 2024)
IPa-2	G6 genus , species cluster A	(Brauer *et al.* 2024)
**Kallioja**	G7 genus , species cluster A	(Brauer *et al.* 2024)
**Kompost-2**	G7 genus , species cluster B, tested with Δ4ϕ	(Brauer *et al.* 2024)
**Kurepalu-1**	G8 genus , species cluster A	(Brauer *et al.* 2024)
**Kurepalu-2**	G9 genus , species cluster A, tested with Δ4ϕ	(Brauer *et al.* 2024)
**Pori-3**	G9 genus , species cluster B, representative phage	(Brauer *et al.* 2024)
Mudajogi	G9 genus , species cluster B	(Brauer *et al.* 2024)

Table S2 Oligonucleotides used in the study

**Name**	**Sequence (5'-3')[a]**	**Purpose**
XbaRelA-For	TTTTTTCTAGATGAGGTGGTGGCAGTGGAAGG	Contruction of pEMGΔ*relA*
RelAdel-Rev	CTTTGTTTTACCTTTCCATGCAGCACAC	Contruction of pEMGΔ*relA*
RelAdelLONG-For	GTGTGCTGCATGGAAAGGTAAAACAAAGACCGGGGCGTCTGAATTCG	Contruction of pEMGΔ*relA*
RelA-EcoRI-Rev	TTTTGAATTCTTCCCAGCGCGACTTGACC	Contruction of pEMGΔ*relA*
XbaSpoT-For	TTTTTCTAGACTACAAGCTACGCGGCCCCTG	Contruction of pEMGΔ*spoT*
SpoTdel-Rev	CATGGGTTCACCTCCTGCCG	Contruction of pEMGΔ*spoT*
SpoTdelLONG-For	CGGCAGGAGGTGAACCCATGCGAAAAGTGCGTGCAACTCTCC	Contruction of pEMGΔ*spoT*
EcoRISpoT-Rev	TTTTGAATTCGAAGGGCAGTGCTTGACGCATG	Contruction of pEMGΔ*spoT*
SpoT IN For	ATGCACATGGACCATCAGAG	Verification of deletion of *relA*
SpoT IN Rev	AAGCCAAGGTCTTCGAACTC	Verification of deletion of *relA*
RelA IN Fw	ATCGCCAAGCTGTTGCATG	Verification of deletion of *spoT*
RelA IN Rev	AAGTTCGGCCTCTTCGTGC	Verification of deletion of *spoT*

[a] Restriction enzyme recognition sequences used for cloning are underlined

Table S3. Descriptive statistics for cell size mesurements

	**PaW85 3 h**	**ΔrelA 3 h**	**ΔrelAΔspoT 3 h**	**PaW85 24 h**	**ΔrelA 24 h**	**ΔrelAΔspoT 24 h**
**N**	681	1718	2158	2046	2299	1589
**Minimum**	1.204	1.178	1.324	1.253	1.18	0.8827
**1st quartile**	3.201	3.46	3.438	2.202	2.07	2.044
**Median**	3.869	4.4	4.417	2.61	2.531	2.617
**3rd quartile**	5.031	5.62	5.601	3.087	3.112	3.582
**Maximum**	9.461	13.79	14.31	9.686	13.52	14.59

Table S4 MIC values for *Pseudomonas putida* wild type PaW85 and its ΔrelA and ΔrelAΔspoT derivatives

**MIC-s (median (range); N≥3)****					
**Class**	**Chemical**	**Concentration unit**	**PaW85 wt**	**PaW85 ΔrelA**	**PaW85 ΔrelAΔspoT**
Chelator	Ethylenediaminetetra-acetic acid (EDTA)	mM	1.6	1.6	1.6
Salt	NaCl	%	7.5	7.5	7.5
DNA damaging agent	Nitroquinoline (NQ)	mM	0.8	0.8	0.8<br>(0.4...0.8)
	Mitomycin C	µg/ml	16<br>(16...32)	16	16
AB*: DNA damage	Ciprofloxacin (Cip)	µg/ml	0.125<br>(0.125...0.25)	0.5<br>(0.25...0.5)	0.125<br>(0.125...0.25)
AB: cell wall damage	Polymyxin B	µg/ml	1	1<br>(1...2)	1
	Benzylpenicillin (Bp)	µg/ml	512<br>(512...1024)	512<br>(512...1024)	512
AB: translation inhibitor	Doxycycline	µg/ml	2	2	2
	Chloramphenicol	µg/ml	128	128	128<br>(128...256)
	Gentamycin (Gm)	µg/ml	8	8	8<br>(16...4)

*AB – antibiotic

** Number in parenthesis marks the range of values from at least three independent experiments

Bayley SA, Duggleby CJ, Worsey MJ *et al.* Two modes of loss of the tol function from *Pseudomonas putida* mt-2. *Molec Gen Genet* 1977;**154**:203–4.

Brauer A, Rosendahl S, Kängsep A *et al.* Isolation and characterization of a phage collection against *Pseudomonas putida*. *Environmental Microbiology* 2024;**26**:e16671.

Martínez-García E, de Lorenzo V. Engineering multiple genomic deletions in Gram-negative bacteria: analysis of the multi-resistant antibiotic profile of *Pseudomonas putida* KT2440: Tools for editing Gram-negative genomes. *Environmental Microbiology* 2011;**13**:2702–16.

Wong SM, Mekalanos JJ. Genetic footprinting with *mariner* -based transposition in *Pseudomonas aeruginosa*. *Proc Natl Acad Sci USA* 2000;**97**:10191–6.
