# Supplementary material for: The opposite effects of stringent response on phage infection of Pseudomonas putida
Source: Microlife. 2026 Jan 2;7:uqaf048. doi: 10.1093/femsml/uqaf048 (PMC12814882; doi:10.1093/femsml/uqaf048)
Supplement: uqaf048_Supplemental_Files [file uqaf048_supplemental_files.zip › Supplementary File 2_Figures_REVISED.pdf]

Supplementary file 2

**Supplementary figures**

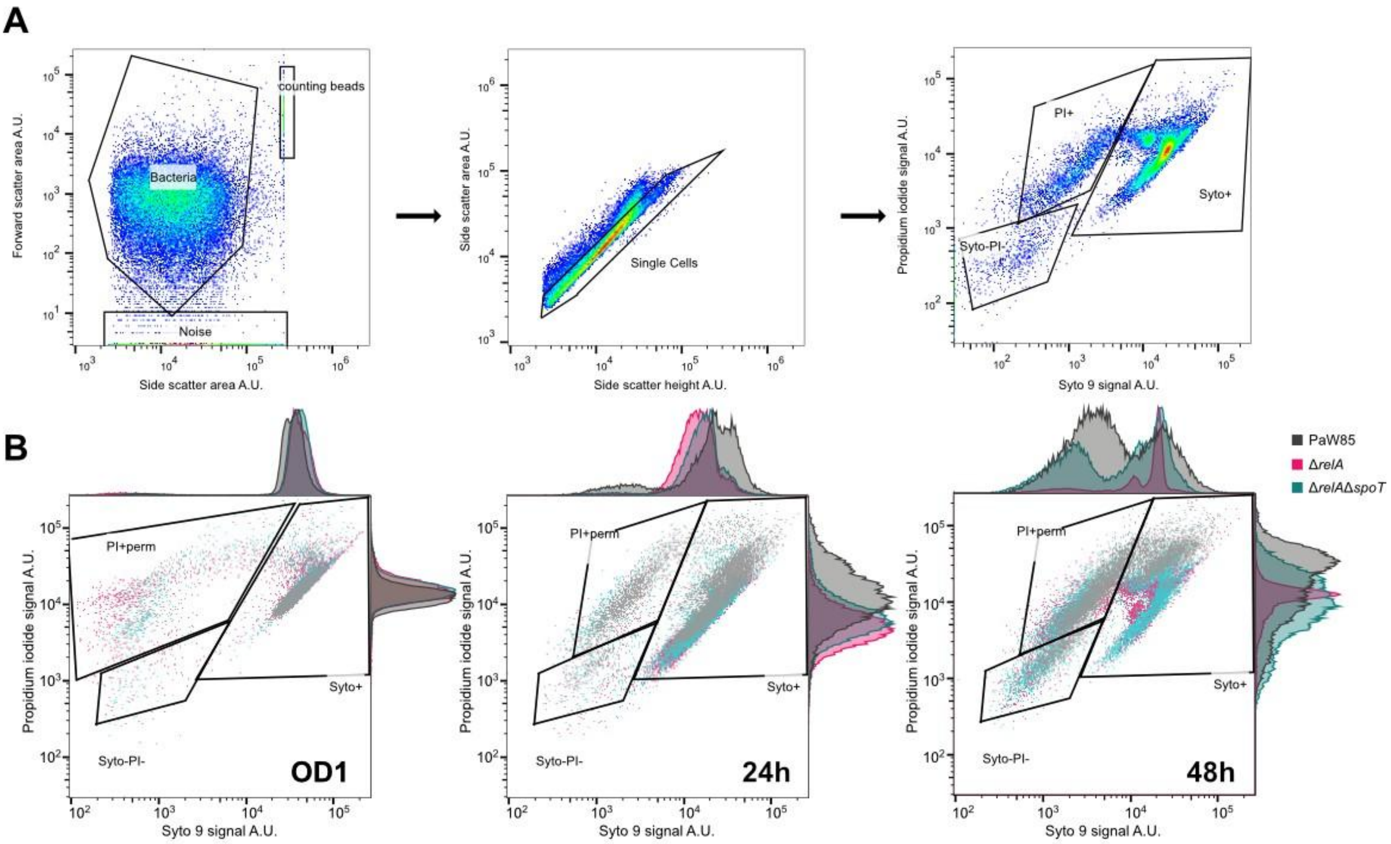


**Figure S1.** Flow cytometry dot plots after staining with propidium iodide (PI) and Syto 9. Representative figures are shown. A. Gating strategy for flow cytometry analysis. Bacteria were selected from forward and side scatter plots, and singlets were selected from side scatter height versus area plots. PI+, Syto+ and Syto-PI- populations were analysed. B. Syto 9 and PI scatter plots of PaW85 wt, ΔrelA and ΔrelAΔspoT after growth in LB medium. Samples have similar fluorescence values during exponential growth (OD1), however differences appear in stationary phase (24 h and 48 h), indicating altered membrane permeability.

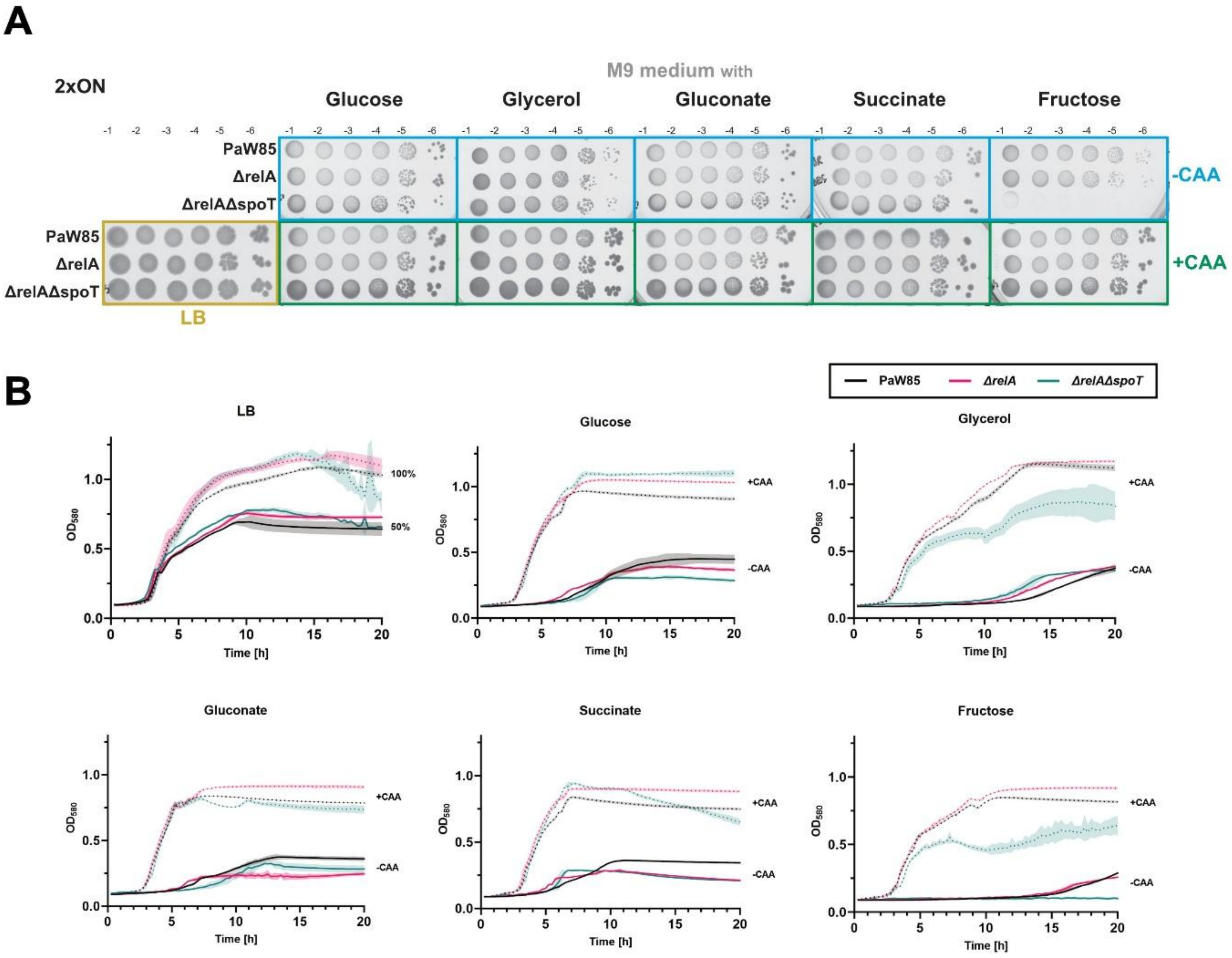


**Figure S2.** Growth on/in minimal media of *Pseudomonas putida* wild type PaW85 and its stringent response deletion derivatives. A. *P. putida* PaW85 and its derivatives ΔrelA and ΔrelAΔspoT ((p)ppGpp$^0$) on M9 minimal medium without amino acids to similar CFU-s to wild type after incubation for 48 hours (2xON). 24-hour cultures were diluted in a series of 10-fold dilutions and 5 µL drops spotted on agar plate. Representative images from experiments with 3 biological replicates. B. Growth in liquid rich or M9 minimal media with (dotted lines) or without (solid lines) casamino acids. Plate reader data of exponential precultures, means±SD, N=3 biological replicates.

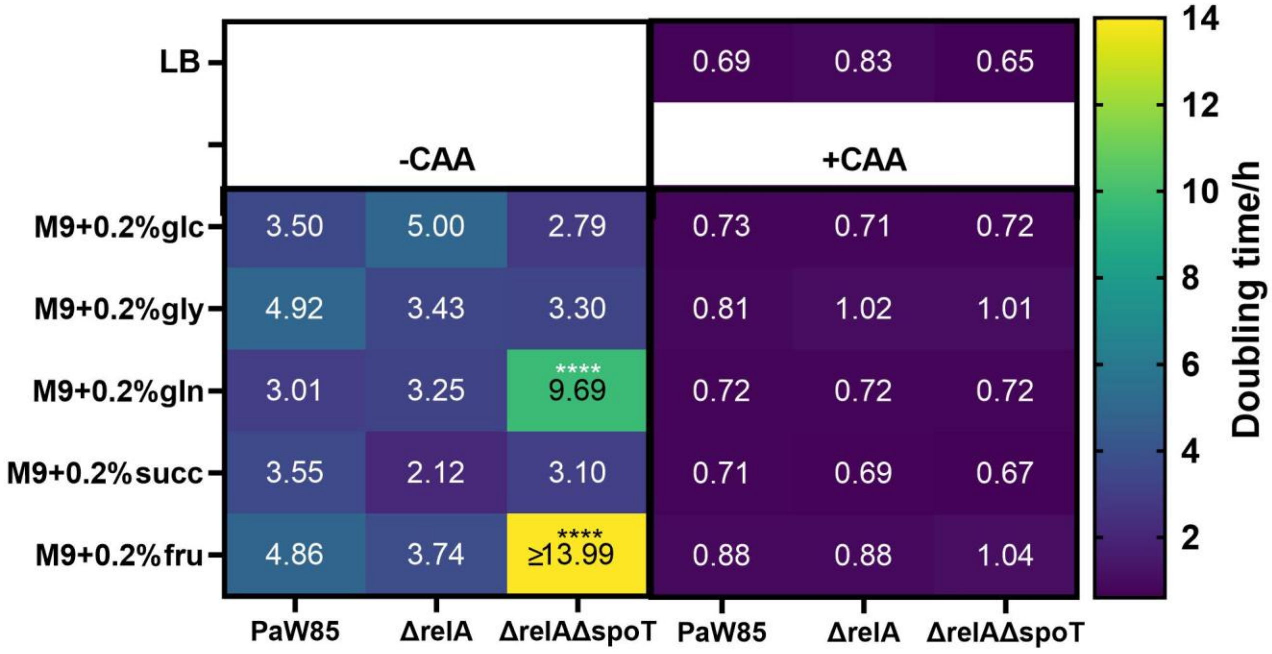


**Figure S3.** Doubling times in LB and M9 minimal media (±CAA) based on microtiter plate optical densities. Means shown (N=3).

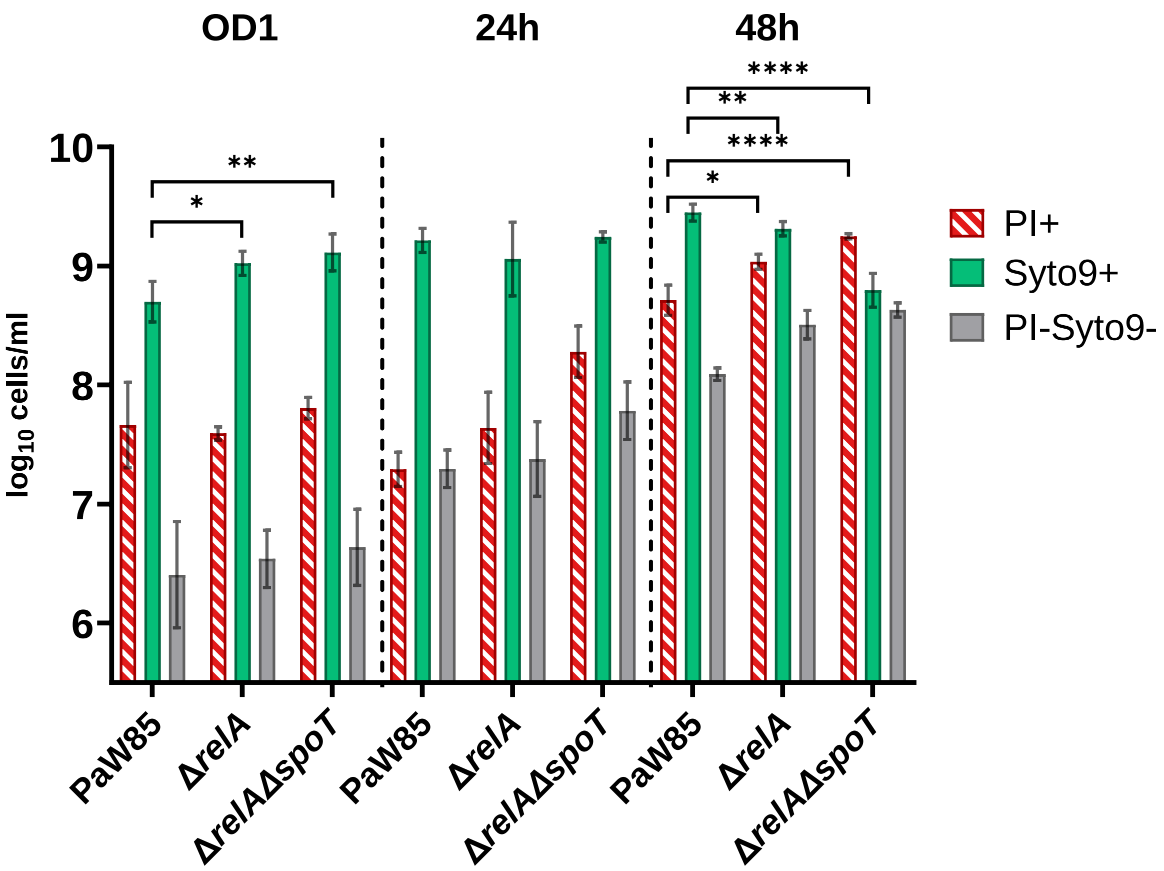


**Figure S4**. Single cell concentrations per millilitre of culture from propidium iodide (PI; red-white striped) and Syto 9 (green) stained cells based on flow cytometry analysis. Counting beads were added to flow cytometry samples to estimate sample volume. Cells were grown in liquid LB at 30 °C before analysis. Ordinary two-way ANOVA with uncorrected Fisher's LSD with a single pooled variance. * p value < 0.05; ** p value < 0.005; *** p value < 0.0005; **** p value < 0.0001

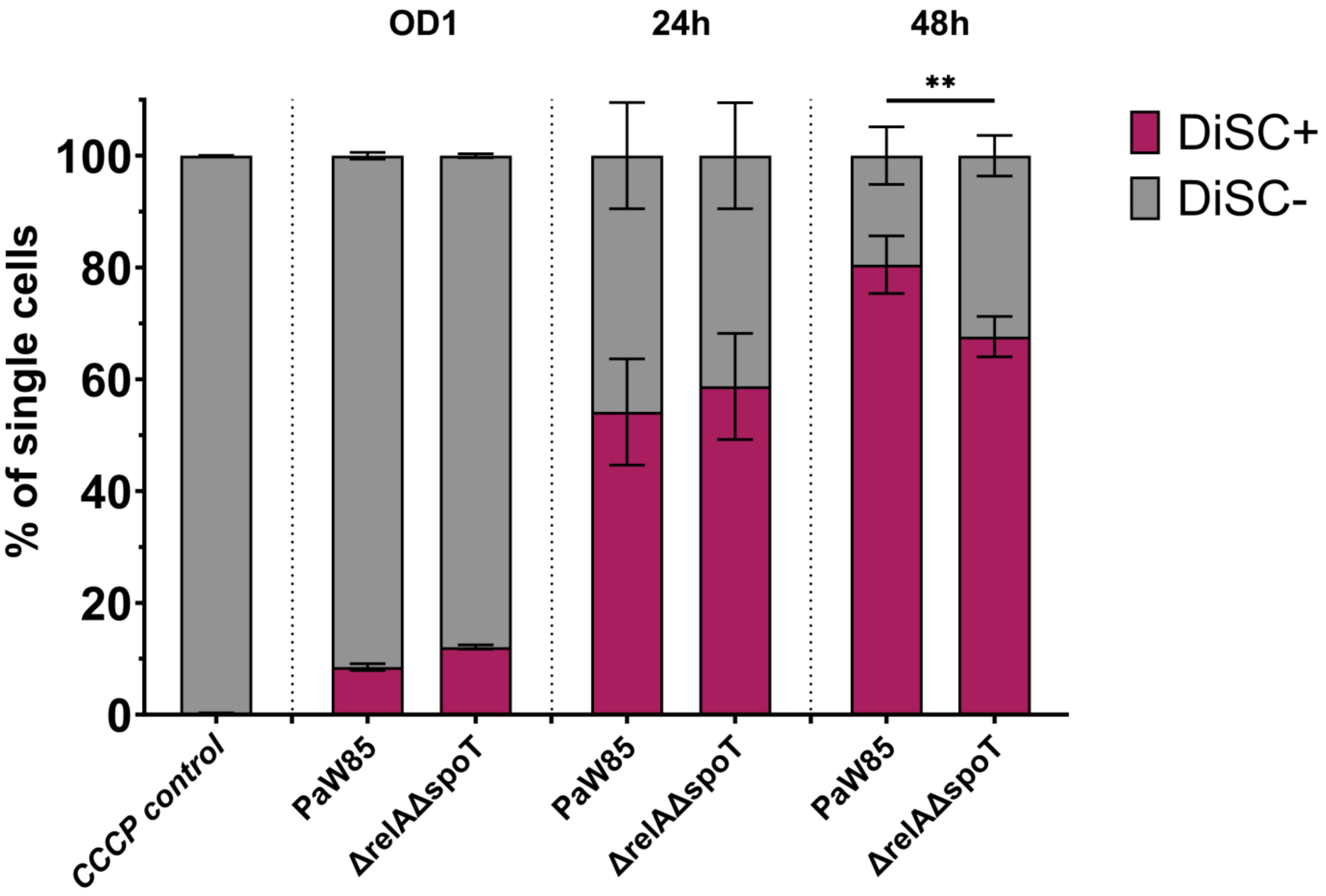


**Figure S5**. Flow cytometry analysis of LB-grown cells stained with membrane potential indicator dye $DiSC_3(5)$. Means±SD (N=3). Ordinary two-way ANOVA with uncorrected Fisher's Least Significant Difference (LSD) test with a single pooled variance. ** - p value <0.005

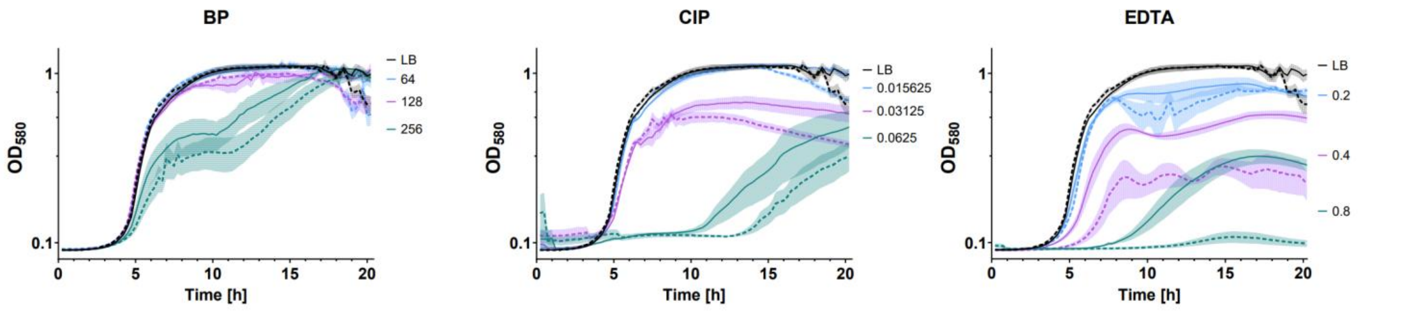


**Figure S6**. Growth curves of wild type PaW85 *P. putida* (solid lines) and ΔrelAΔspoT ((p)ppGpp$^0$, dashed lines) with benzylpenicillin (BP), ciprofloxacin (CIP), and ethylenediamine tetraacetate (EDTA). Number indicates concentration of chemical in µg/mL (BP, CIP) or mM (EDTA). Initial inoculum was $10^5$ exponential phase cells/mL and cells were grown at 30 °C with shaking in a microtiter plate reader. Data show mean±SD (N = 3 biological replicates, each in 3 technical replicates).

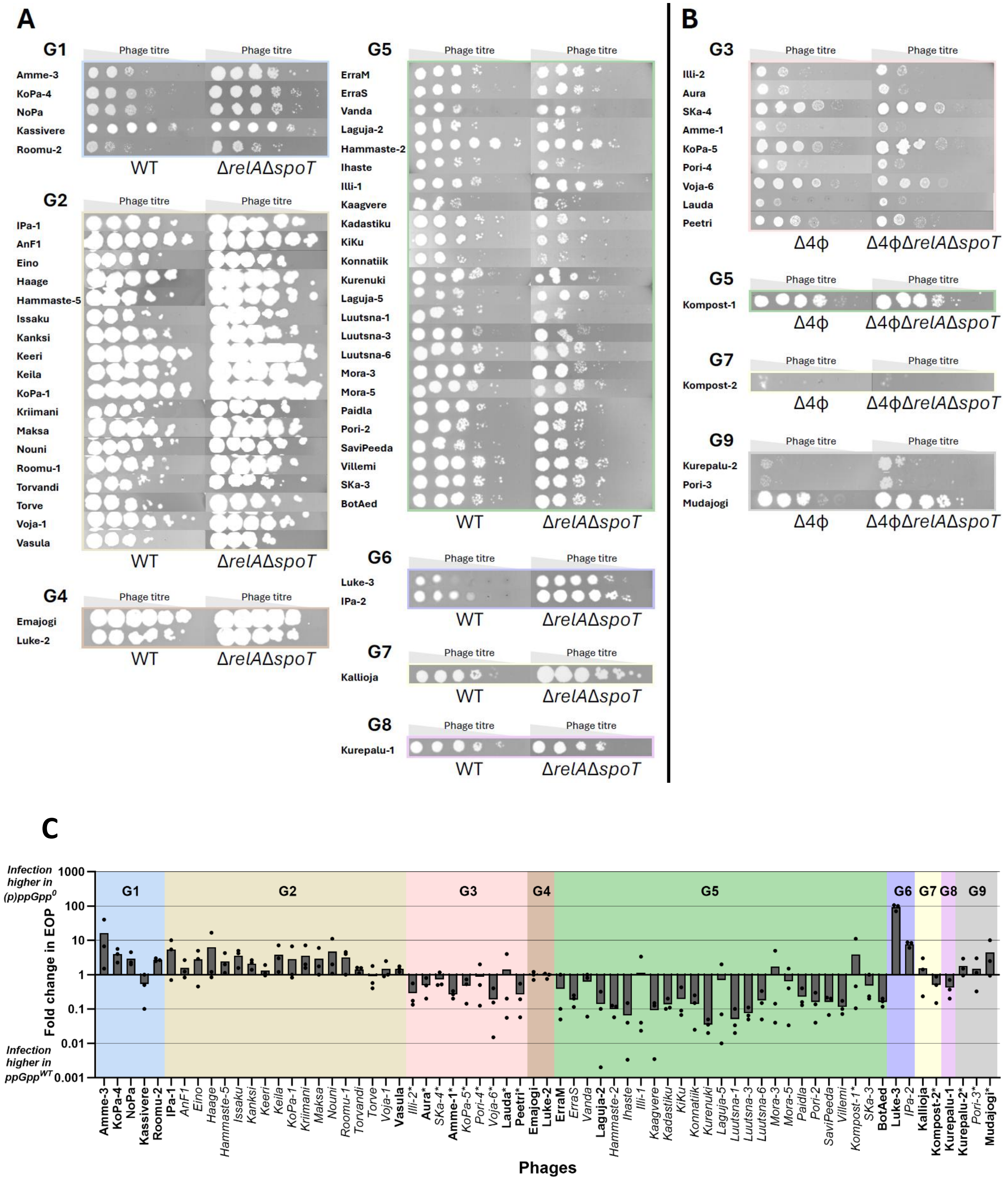


**Figure S7.** Stringent response has opposite effects on the infection of CEPEST. A and B. Representative pictures of phage infection efficiency dependence on stringent response in wild type background (A) PaW85 (WT) compared to ΔrelAΔspoT or prophage free background (B) Δ4ϕ compared to Δ4ϕΔrelAΔspoT. C. Fold change in phage plaque counts in the absence of stringent response (PFU $ppGpp^{wt}$/PFU $ppGpp^{0}$) for all CEPEST phages.

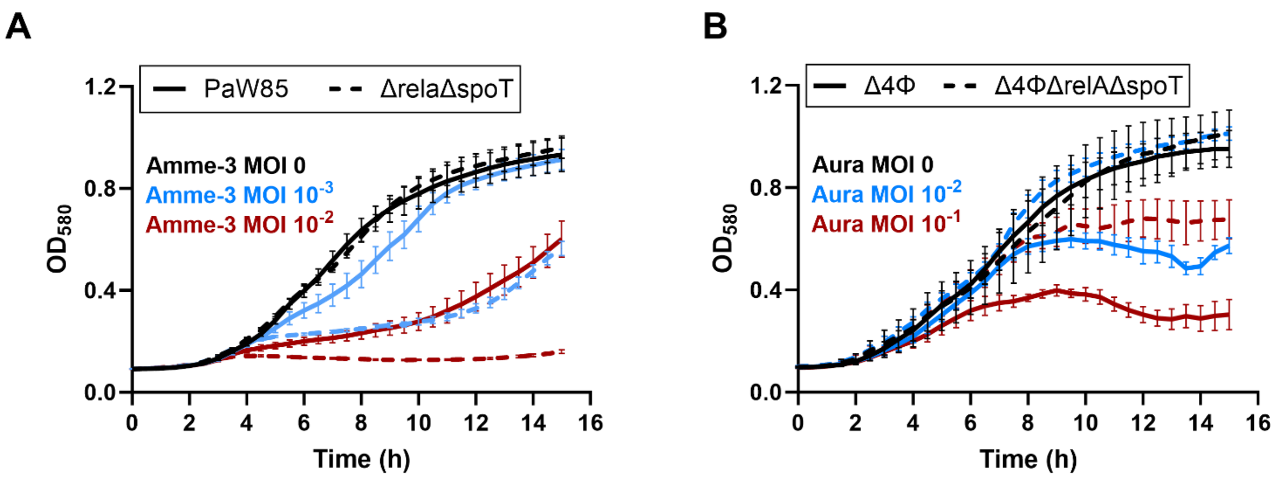


**Figure S8.** Stringent response effect on phage infection in liquid media. A. Growth of *P. putida* PaW85 (solid line) and its ΔrelAΔspoT derivative (dashed line) at 20 °C without infection (black) or with Amme-3 phage at MOI values of $10^{-3}$ (blue) and $10^{-2}$ (red). B. Growth of prophage free *P. putida* PaW85 Δ4ϕ (solid line) and its (p)ppGpp$^0$ derivative (Δ4ϕΔrelAΔspoT, dashed line) at 20 °C without infection (black) or with Aura phage at MOI values of $10^{-2}$ (blue) and $10^{-1}$ (red). The values represent means of three independent experiments with all at least six technical replicates. Whiskers show 95% CI.

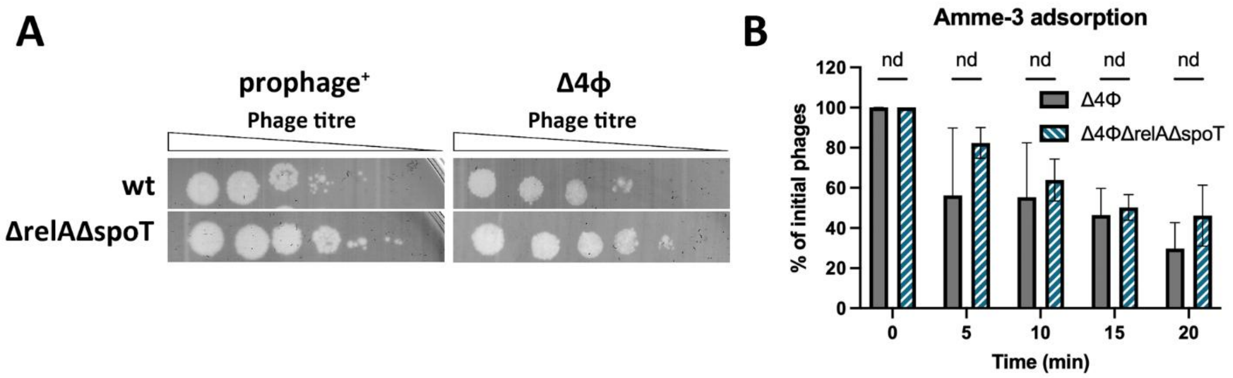


**Figure S9.** Amme-3 infection in prophage free background does not change ppGpp effect. A. Representative pictures of Amme-3 infection efficiency dependence on stringent response in wild type background (prophage$^+$) or prophage free background (Δ4ϕ) compared to their (p)ppGpp$^0$ derivatives (ΔrelAΔspoT). B. Infectious Amme-3 phages remaining in the supernatant after adsorption to prophage free *P. putida* PaW85 (Δ4ϕ) and its ppGpp$^0$ derivative (Δ4ϕΔrelAΔspoT). Means±95% confidence intervals (N≥3) are shown. Data was analysed using multiple unpaired t-test with a two-stage step-up method of Benjamini, Krieger and Yekuteli, nd - non-discovery.
